# The pioneer factor SOX9 competes for epigenetic factors to switch stem cell fates

**DOI:** 10.1038/s41556-023-01184-y

**Published:** 2023-07-24

**Authors:** Yihao Yang, Nicholas Gomez, Nicole Infarinato, Rene C. Adam, Megan Sribour, Inwha Baek, Mélanie Laurin, Elaine Fuchs

**Affiliations:** 1 Howard Hughes Medical Institute, Robin Chemers Neustein Laboratory of Mammalian Cell Biology and Development, The Rockefeller University, New York, NY USA; 2 Present Address: Allen Institute for Cell Sciences, Seattle, WA USA; 3 Present Address: PRECISIONscientia, Yardley, PA USA; 4 Present Address: Regeneron Pharmaceuticals, Tarrytown, NY USA; 5 Present Address: Kyung Hee University, Seoul, South Korea; 6 Present Address: CHU de Québec-Université Laval Research Center, Quebec City, Quebec Canada

**Keywords:** Chromatin remodelling, Skin stem cells, Reprogramming

## Abstract

During development, progenitors simultaneously activate one lineage while silencing another, a feature highly regulated in adult stem cells but derailed in cancers. Equipped to bind cognate motifs in closed chromatin, pioneer factors operate at these crossroads, but how they perform fate switching remains elusive. Here we tackle this question with SOX9, a master regulator that diverts embryonic epidermal stem cells (EpdSCs) into becoming hair follicle stem cells. By engineering mice to re-activate SOX9 in adult EpdSCs, we trigger fate switching. Combining epigenetic, proteomic and functional analyses, we interrogate the ensuing chromatin and transcriptional dynamics, slowed temporally by the mature EpdSC niche microenvironment. We show that as SOX9 binds and opens key hair follicle enhancers de novo in EpdSCs, it simultaneously recruits co-factors away from epidermal enhancers, which are silenced. Unhinged from its normal regulation, sustained SOX9 subsequently activates oncogenic transcriptional regulators that chart the path to cancers typified by constitutive SOX9 expression.

## Abstract (web summary)

Yang, Gomez et al. show that the pioneer factor SOX9 regulates the switch from epidermal stem cell to hair follicle stem cell fate by binding and opening hair follicle enhancers, while recruiting epigenetic factors away from epidermal enhancers.

## Main

From development to malignancy, cells face decisions of fate determination. Governing the reprogramming from one fate to another, pioneer factors are transcription factors that can recognize and access their cognate binding motifs in compacted and repressed chromatin^1^. In vitro studies have shown that when a pioneer factor binds, it displaces the nucleosome, permitting the opening and remodelling of the chromatin landscape to change gene expression^2,3^. Recent studies have begun to uncover interactions of various pioneer factors with histone-modifying enzymes and members of the SWI/SNF chromatin remodelling complex^2^. However, the order of events in chromatin remodelling has remained elusive due to the rapid time frame of reprogramming in vitro where cells are outside local restraints of their tissue microenvironments. Even less clear is the role of pioneer factors in accomplishing the other side of fate switching, namely the silencing of a cell’s previous identity^2^.

In this Article, seeking the answers to these enigmas, we focused on the SOX superfamily of context-specific pioneer factors, whose members are at the nexus of critical cell fate choices in embryonic development, tissue homeostasis and transition to malignancy^4–7^. In skin, SOX9 is first expressed when multipotent embryonic epidermal progenitors bifurcate to become SOX9^+^ hair follicle stem cells (HFSCs) and SOX9^neg^ epidermal stem cells (EpdSCs)^8–10^. In the next step of hair follicle morphogenesis, SOX9^+^ HFSCs bifurcate again to form SOX9^neg^ transit amplifying hair shaft progenitors. Basal cell carcinoma (BCC) formation from EpdSCs resembles the initial steps of embryonic hair follicle morphogenesis, but once re-activated, SOX9 is sustained, leading to invaginating follicle-like tumour masses that lack hair lineages^11–14^. Here we recapitulated these reprogramming events by generating mice in which we could inducibly re-activate and sustain SOX9 expression in adult EpdSCs.

Not encountered in vitro or in embryogenesis, the mature tissue stem cell niche imposed physiological constraints that slowed SOX-mediated chromatin reprogramming. This enabled the unravelling of sequential events that happen as SOX9 achieves a cell fate switch that when dysregulated later progresses to a tumourigenic state. By dissecting the temporal steps of epigenetic reprogramming, we show that SOX9 binds to closed chromatin at HFSC enhancers, where it recruits histone and chromatin modifiers to remodel and subsequently open chromatin for transcription. In doing so, SOX9 redistributes co-factors away from EpdSC enhancers, thereby silencing these genes indirectly but efficiently. Moreover, when the ability of SOX9 to bind DNA is abrogated, it still silences, but when it cannot bind chromatin remodellers, the switch fails altogether. Together, our findings illuminate how fate switching can be achieved through the direct activating functions of a pioneer factor, which then unleashes transcriptional repression through indirect competition for epigenetic co-factors. We further show that SOX9 regulates downstream transcription factors to drive tumourigenesis, which explains the delay in subsequent reprogramming events.

## Results

### SOX9 launches a transcriptional cascade towards BCC in EpdSC

**Extended Data Fig. 1:**
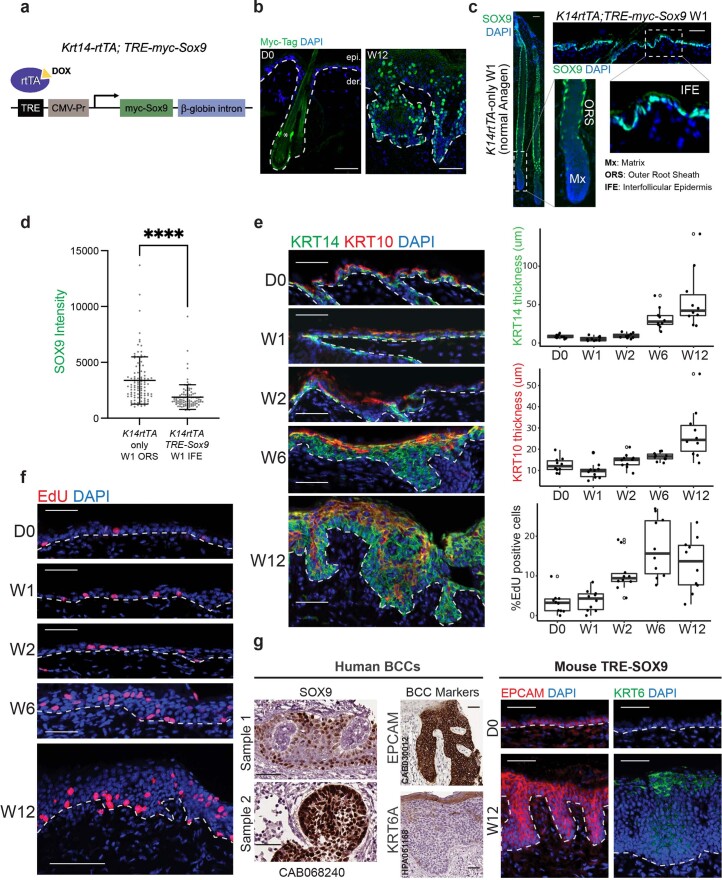
Ectopic reactivation of SOX9 in adult EpdSCs silences epidermal fate and activates hair follicle fate within 2W and then progresses to BCC-like lesions. **a**, Schematic of the *TRE-Sox9* construct for generating SOX9-inducible transgenic mice. **b**, Immunofluorescence showing myc-tag staining at D0 and W12 SOX9-induction. This validates myc-tag expression is only induced and specifically induced in EpdSCs. All scale bars are 50μm. **c**, Immunofluorescence comparing SOX9 expression in the normal anagen ORS (containing activated HFSCs) and induced SOX9 expression in the adult EpdSCs. All scale bars are 50μm. **d**, Quantifications of SOX9 immunofluorescence intensity in the two states in (b) shows that SOX9 levels in ectopically induced adult EpdSCs are not higher than in native ORS HFSCs. n = 100 cells measured over 5 biological replicates. All the error bars are mean ± SD. Statistical significance from two-tailed t-test is denoted by ****(p < 0.0001). **e**, (left) KRT14 and KRT10 immunofluorescence of the skin after SOX9-induction in EpdSCs. Note that the KRT14 skin progenitor is markedly expanded over time. All scale bars are 50μm. (right) Quantification of the thickness of KRT14 and KRT10 layers over time. n = 5 biological replicates with 2 measurement per sample. Boxplots are centered at median and bound by 1st and the 3rd quartile, and whiskers extend to 1.5 times IQR on both ends. The solid dots are data points, and the empty circles are outliers beyond 1.5 times IQR. **f**, (top) cell proliferation as assessed by EdU immunofluorescence after SOX9-induction. All scale bars are 50μm. (bottom left) quantification of % proliferating cells in the basal layer of EpdSCs. **g**, (left) Immunostaining of human BCC samples from The Human Protein Atlas. SOX9 and EpCAM show strong staining in the body of the lesion, whereas KRT6A is restricted to the apical epidermis. (right) Immunofluorescence images show the lesions in 12-week samples have similar EpCAM and KRT6 staining pattern as human BCC. Dotted lines denote the dermo-epidermal border. All scale bars are 50μm. Source data

To interrogate SOX9 reprogramming in adult tissue stem cells, we engineered mice harbouring a MYC-epitope-tagged *Sox9* transgene controlled by a tetracycline responsive enhancer and a minimal promoter (*TRE-Sox9*) (Extended Data Fig. 1a). After validating the specificity of transgene induction (Extended Data Fig. 1b), we bred selected mice to lines expressing the requisite tetracycline-inducible transcriptional activator (rtTA) driven by an epidermal (*Krt14*) promoter (*Krt14-rtTA*)^15^, and selected mice that induced MYC–SOX9 in EpdSCs at levels comparable to SOX9 in adult HFSCs (Extended Data Fig. 1c,d).

**Fig. 1:**
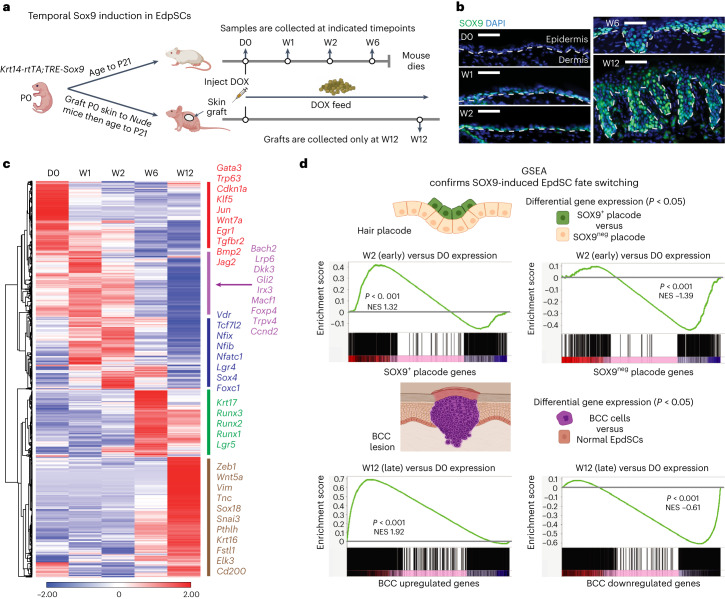
In EpdSCs of adult skin, sustained SOX9 re-activation silences the epidermal while activating the hair follicle and subsequently BCC fates. **a**, Schematic of SOX9 induction in EpdSCs of adult and post-engrafted skin. **b**, Immunofluorescence reveals the appearance of nuclear SOX9 in EpdSCs after DOX induction. Dotted lines denote epidermal–dermal borders. Scale bars, 50 μm. **c**, Heat map of temporal RNA-seq data shows significantly variable genes (DESeq2 Wald test, adjusted *P* value <0.05) across combined independent replicates (*r* > 0.94) of each of five indicated timepoints. Hierarchical clustering revealed five distinct patterns, shown as coloured bars on the right with representative transcripts indicative of specific fates. **d**, GSEA of W2 versus D0 SOX9-induced expression changes in adult EpdSCs compared with SOX9^+^ and SOX9^neg^ wild-type placode gene signatures from embryonic day E15.5 (top) (Kolmogorov–Smirnov test, *P* < 0.001 for both gene sets), and W12 versus D0 SOX9-induced EpdSC expression changes compared to BCC upregulated and downregulated signatures (bottom) (Kolmogorov–Smirnov test, *P* < 0.001 for both gene sets). NES, normalized enrichment score.

Upon doxycycline (DOX) administration (D0), adult mice were monitored weekly thereafter (Fig. 1a). Within the first 2 weeks, morphology and differentiation seemed unaffected (Extended Data Fig. 1e). However, by week (W)1, nuclear SOX9 was detected in the EpdSCs of the innermost (basal) epidermal layer (Fig. 1b). By W2, a rise in proliferation was detected, similar to that seen when SOX9 is naturally induced in embryonic epidermis^10^ (Extended Data Fig. 1f).

Between W2 and W6, de novo invaginations began to grow between native HFs (Fig. 1b and Extended Data Fig. 1e). As differentiation defects necessitated killing mice by W6, we monitored later events in SOX9 reprogramming by engrafting neonatal *Krt14-rtTA;TRE-Sox9* skin patches onto immunocompromised mice. Once normal skin pathology was restored (21 days after grafting), we induced SOX9 (Fig. 1a). By W12 post-induction, invaginations were dysplastic, resembling morphological and molecular (SOX9, EpCAM and KRT6) features of human BCCs (Extended Data Fig. 1g).

**Extended Data Fig. 2:**
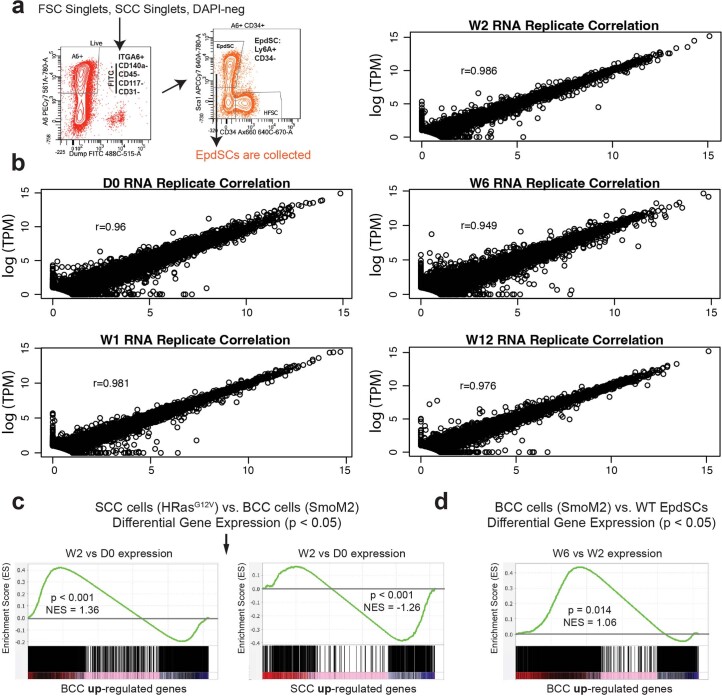
Quality control for transcriptome analysis and temporal comparisons between SOX9-induced transcriptome analyses and cutaneous cancers. **a**, FACS gating strategies for isolating EpdSCs. DAPI-negative singlets were first gated on ITGA6 (epithelial progenitors) and immune cell, fibroblast/adipocyte, endothelial and melanocyte lineage markers (CD45, CD140a, CD31 and CD117). Lineage-negative and ITGA6^+^ cells were further gated on CD34 and Ly6A/E (SCA-1) to distinguish HFSCs and EpdSCs, respectively. **b**, Replicate correlation analyses of RNA-seq show strong correlation (r > 0.94) between samples across time points. **c**, At W2 following SOX9 induction, similarities to BCC were already apparent (K-S test, p < 0.001). By contrast, a negative correlation to SCC was observed (K-S test, p < 0.001). **d**, In comparing all BCC genes to our W6 and W2 data, the shift towards a BCC signature continued to rise, concomitant with the phenotypic changes described in Fig. 1 (K-S test, p = 0.014).

To gain further insights, we profiled the transcriptomic changes occurring in EpdSCs during SOX9-driven reprogramming. At each timepoint, two biological replicates of RNA sequencing (RNA-seq) were performed on fluorescence-activated cell sorting (FACS)-purified EpdSCs from *Krt14-rtTA*;*TRE-Sox9* skins (Extended Data Fig. 2a,b). By comparing transcriptomes across time, we identified the significantly variable genes (*P* < 0.05) along the reprogramming cascade (Fig. 1c and Supplementary Table 1). As expected, the D0 population displayed the hallmark signature of EpdSCs, replete with mRNAs encoding epidermal master regulator transcription factors, TRP63 and GATA3, key signalling pathways (NOTCH and EGFR), and epidermal structural proteins.

Despite few morphological changes within 2 weeks after induction, SOX9^+^ EpdSCs displayed dramatic transcriptional changes, mimicking transcriptional changes that occur when embryonic skin progenitors naturally induce SOX9 and divert from an epidermal to hair follicle fate^10^. Thus, epidermal genes were markedly suppressed, while classical markers of the embryonic hair bud and adult hair follicle outer root sheath (ORS) were upregulated, as supported by gene set enrichment analysis (GSEA) (Fig. 1c,d). The kinetics of these reprogramming events in adult EpdSCs, however, was markedly slower in the adult than in embryonic skin or in cultured cells, suggestive of the need to override the constraints of the mature epidermal niche.

As in BCC development, progression to mature HFs did not happen, in agreement with the need for *Sox9* downregulation for HFSCs to generate the hair and its channel^10,16^. However, with sustained SOX9 expression, the transcriptional changes continued, and by W6–12, cancer-associated features appeared. At W12, GSEA revealed a strong correlation, both up and down, with the molecular signature of BCC compared with normal skin^14,17^ (Fig. 1c,d). Although the similarities in gene expression were strongest at late stages, they surfaced as early as W2, that is, before overt phenotypic changes, and clearly favoured a BCC versus squamous cell carcinoma (SCC) signature (Extended Data Fig. 2c,d).

### SOX9 is a bona fide pioneer factor

**Fig. 2:**
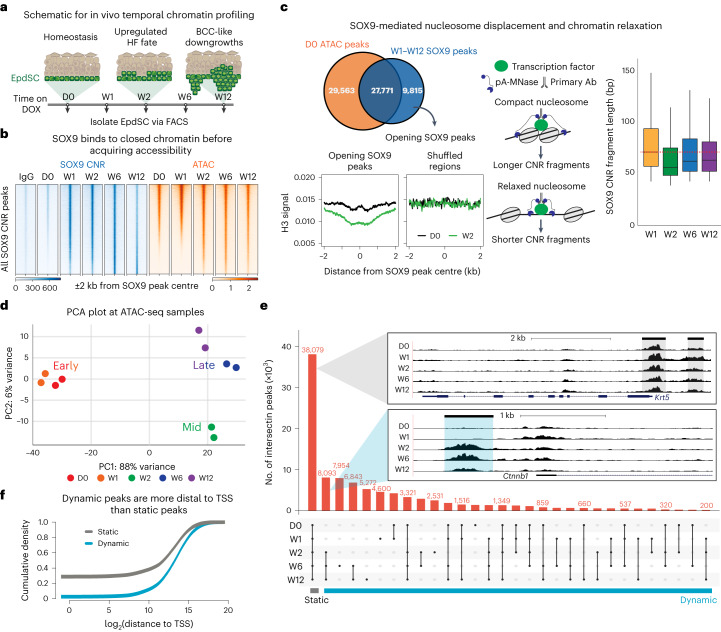
Upon induction, SOX9 opens chromatin at enhancers by evicting the nucleosome at its binding site and remodelling the flanking chromatin. **a**, Schematic of morphological changes that occur temporally after DOX. Back skins were collected at indicated timepoints and subjected to EpdSC FACS purification followed by SOX9 CNR landscaping and ATAC-seq. **b**, Heat map of IgG control CNR (blue), SOX9 CNR (blue) and ATAC-seq (orange) signals at all SOX9-bound peaks across indicated timepoints. Peaks are arranged along the vertical axis on the basis of their accessibility at W1. Note that, within the cohort of peaks in the bottom half along this axis, SOX9 binding occurred by W1, while their ATAC-seq landscape did not change until the following week. **c**, Top left: Venn diagrams show that, although SOX9 binds to many pre-existing accessible chromatin peaks, 9,815 SOX9 peaks open de novo. MINT-ChIP for histone H3 (H3) coupled with SOX9 CNR reveals that, by W2 post-induction, SOX9 binds to these previously closed peaks, concomitant with displacement of histone H3 at the SOX9-bound site. Right: schematic and data showing that CNR fragment length shortens between W1 and W2, correlating with SOX9 binding and nucleosome eviction. *n* = 2 biological replicates. Box plot is centred at median and bound by first and the third quartile, and whiskers extend to 1.5 times interquartile range (IQR) on both ends. **d**, PCA of ATAC-seq duplicate samples over the five timepoints. **e**, Upset plot of ATAC-seq peaks for each timepoint following SOX9 induction. Shared regions between timepoints are indicated by the dots and connecting lines. Grey inset shows a genome browser track of the generic skin stem cell *Krt5* locus as an example of a static gene region. Blue inset depicts a dynamic region of the WNT-target HFSC gene *Ctnnb1* that is open by W2 and remains open across W6 and W12 timepoints. **f**, Empirical cumulative distribution plot of dynamic (blue) and static peaks (grey) and their density relative to the transcription start site (TSS) of the nearest gene. Note that dynamic peaks are primarily associated with distal regions, typically encompassing enhancers.

**Extended Data Fig. 3:**
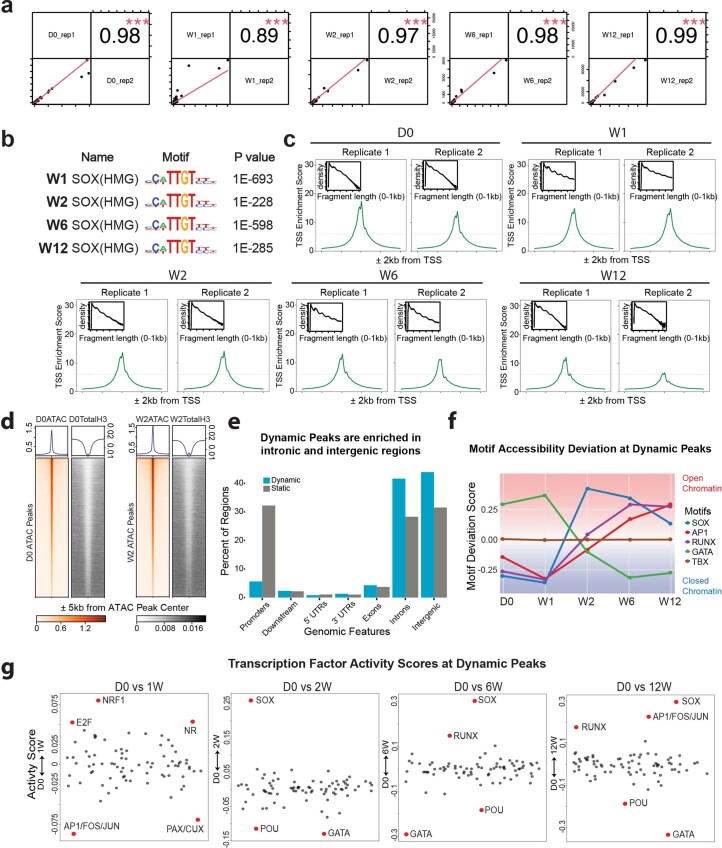
Analyses and dynamics of ATAC-seq and Cut-and-Run and peak distributions. **a**, Replicate correlation analyses of SOX9 Cut-and-Run show strong correlation between duplicate samples at each time point. **b**, HOMER motif analysis shows that SOX(HMG) motifs are strongly enriched in SOX9 CNR peaks in W1 to W12 samples. **c**, ATAC-seq samples show clear nucleosome patterning and TSS enrichment. **d**, Total histone H3 signals are mutually exclusive from ATAC signals at D0 and W2 time points. **e**, Distribution of dynamic and static peaks at different genomic features. Dynamic peaks are more enriched in intronic and intergenic regions, while static peaks are more enriched at promoters. **f**, ChromVAR analysis of motif deviation scores of SOX (blue), AP1/FOS/JUN (red), RUNX (purple), GATA (green), and TBX (brown) motifs at indicated time points. Note that SOX motif accessibility rises markedly in dynamic peaks that open within the first two weeks post SOX9 induction while GATA motif accessibility declines. AP1 and RUNX motif accessibility rise between W2 and W6. TBX motif is shown as a control which does not change accessibility overtime. **g**, HINT-ATAC footprint analysis shows how transcription factor (TF) motif footprints differ in activity score at indicated time points compared to D0. Motifs indicated with red dot show significant changes in activity score over D0.

To understand how SOX9 acts as a master regulator to induce these transcriptional dynamics, we began by performing CUT&RUN (cleavage under targets and release using nuclease; hereafter termed CNR) sequencing^18,19^ to temporally assay the binding of SOX9 to chromatin, and assay for transposase-accessible chromatin with high-throughput sequencing (ATAC-seq)^20,21^ to interrogate chromatin accessibility during reprogramming (Fig. 2a,b). Biological replicates were concordant, and the SOX motif was most enriched in our SOX9 CNR peak sets (Extended Data Fig. 3a–c).

SOX9 binding to chromatin occurred rapidly within W1 and before the rise in proliferation. In contrast, the increase in accessibility at SOX9-binding sites occurred between W1 and W2, indicating that SOX9 can bind to closed chromatin (Fig. 2a). In fact, of all the SOX9 CNR peaks pooled from W1 to W12, nearly 30% were situated within closed chromatin at D0 (Fig. 2c). Moreover, by W2, nucleosome occupancy was lost at these sites as measured by histone H3 (Fig. 2c). Additionally, these SOX9-bound opening peaks displayed a time-dependent decrease in CNR fragment length. These features are hallmarks of nucleosome displacement and pioneer factor activity^22^, providing compelling evidence that SOX9 in skin EpdSCs binds to its cognate motifs within closed chromatin, and subsequently perturbs nucleosomes.

### SOX9 induces global chromatin changes at distal enhancers

Since SOX9 bound to closed chromatin at W1, and presumptive nucleosome loss occurred soon thereafter, these events seemed unlikely to account fully for the tumourigenic transcriptional dynamics (Fig. 1c). Probing deeper, we examined how ATAC peaks and their associated genes changed over time.

Principal component analysis (PCA) of chromatin accessibility showed clustering according to times post-SOX9 induction (Fig. 2d). D0 and W1 samples clustered closely, W2 constituted an intermediary, and later timepoints (W6 and W12) made a second cluster. Comparative analyses across all timepoints revealed that many ATAC peaks were shared across samples, reflective of housekeeping genes and/or genes common to both EpdSCs and HFSCs (for example, *Krt5*) (Fig. 2e). By contrast, other ATAC peaks exhibited strikingly dynamic behaviour (for example, WNT-target *Ctnnb1*), indicative of SOX9-induced temporal chromatin remodelling. These dynamic changes were particularly striking at W2 after SOX9 induction (Fig. 2e). Of the peaks that opened by W2, many persisted thereafter.

Upon binning peaks as either static (present at all timepoints, *n* = 38,079) or dynamic (absent in at least one timepoint, *n* = 62,626), it was clear that dynamic peaks were substantially more enriched in distal intergenic regions than static peaks (Fig. 2f and Extended Data Fig. 3e), suggesting a special role for SOX9 in eliciting chromatin changes at enhancers.

### Direct and indirect chromatin remodelling induced by SOX9

**Fig. 3:**
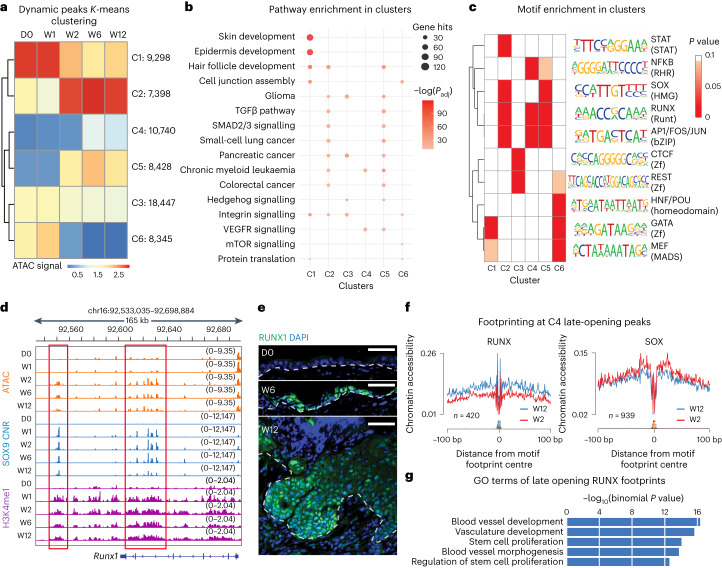
SOX9 triggers an activated HFSC fate, while its target gene transcription factors trigger BCC transformation. **a**, Heat map of *K*-means clustering of ATAC peaks based on signal across time. Number of peaks in each cluster are indicated on the right. **b**, GO-term analysis of the genes associated with each peak of a cluster. Size of circle reflects the number of gene hits for each pathway, while the shade of red indicates adjusted *P* value (*P*_adj_) from binomial test. Note that cluster C1, whose chromatin is suppressed by W2, is enriched for epidermal genes, while C2, whose chromatin is accessible by W2, is enriched for hair follicle genes. C4, whose chromatin is accessible at later times, is the cluster most enriched for BCC genes. **c**, Motif enrichment analysis of each cluster with HOMER. Shade of red indicates *P* value with white representing ≥0.01 (binomial is used to calculate the significance). Note enrichment for: GATA motif in epidermal genes whose chromatin closes after SOX9 induction; SOX motif in hair follicle genes whose chromatin opens early; and the SOX9 target RUNX1 motif in genes whose chromatin opens weeks after SOX9 has bound. **d**, Chromatin landscape of the *Runx1* gene locus, showing that pioneer factor SOX9 binds to this gene (blue), concomitant with early increases in H3K4me1 modifications across the locus (purple), while chromatin accessibility (orange) comes afterwards. Red boxes indicate regions that are bound by SOX9, primed at W1 and opened at W2. **e**, RUNX1 immunofluorescence reveals its absence in epidermal homeostasis, but its presence at late timepoints (W6 and W12) following SOX9 induction. Scale bars, 50 μm. **f**, RUNX and SOX ATAC footprint analyses at W12 and W2, showing a late increase in chromatin accessibility at the RUNX footprint at a stage when phenotypic BCC-like invaginations are prevalent. By comparison, SOX footprints appear by W2 and are retained thereafter. **g**, GO terms of genes whose putative enhancers have RUNX footprints and open at W6 and W12 (binomial is used to calculate the significance).

*K*-means clustering of the dynamic ATAC peaks resolved the temporal changes in chromatin accessibility following SOX9 induction. Although more than 10,000 peaks (cluster C4) opened at later timepoints (Fig. 3a), the most substantial changes occurred between W1 and W2. Using Genomic Regions Enrichment of Annotations Tool (GREAT), we assessed the biological pathways associated with each of the six clusters.

C1 and C6 closed within the first 2 weeks and were enriched for pathways with direct importance to EpdSCs (Fig. 3b and Supplementary Table 2). By contrast, C2 and C5 markedly increased their chromatin accessibility during this time and were enriched for hair follicle development and SHH signalling, key not only in stimulating ORS/HFSC lineage proliferation^23–25^ but also in driving BCCs^11,12,14^ (Fig. 3b). Also notable was a downregulation of AP1, EGFR and TGFβ signalling pathways, which are known to be elevated in BCCs that develop resistance to SHH inhibitors^26^. Many of the pathways enriched in the C2/C5 clusters were also implicated in other cancers previously associated with SOX9 expression^27–29^.

The role of SOX9 in activating the ORS/HFSC fate appeared to be direct, as chromatin that opened by W2 was associated with hair follicle development and displayed both SOX motifs and SOX9 binding (Fig. 3b,c). These peaks also persisted in an accessible state and were still prominent from W6 to W12 (Fig. 3a). By contrast, late-opening C4-associated genes were not related to HFSC fate. Many of their peaks only entered a more accessible state sometime after W2, coincident with the late BCC gene induction per our transcriptome analysis (Fig. 1c,d). Intriguingly, these peaks were not enriched for SOX but rather RUNX, AP1 and NF-κB motifs (Fig. 3c). Moreover, whereas the *Sox9* transgene was induced by W1, *Runx1–Runx3* in particular were highest at W2-W6 (Fig. 1b,c). Given that RUNX1 suppresses basosquamous features in therapeutic-resistant human BCCs^30^, the sustained *Runx* expression underscored a BCC-like rather than SCC-like phenotype.

We also performed temporal motif analysis with ChromVAR^31^, which considers both enrichment and chromatin accessibility variability at each motif. In addition to SOX, AP1(FOS/JUN), GATA and RUNX were top variable motifs. To learn how motif accessibility varied over time, we plotted accessibility deviation scores for each timepoint and compared them with a motif (TBX) that showed no temporal variability. Agreeing with motif enrichments in C4, the RUNX motifs continued to gain accessibility from W2 to W6 (Fig. 3c and Extended Data Fig. 3f).

Delving deeper, the *Runx1* gene locus was closed at D0, but within W1 after induction, the locus revealed SOX9 binding at multiple sites (Fig. 3d). Since the dynamic peaks were enriched at distal intergenic regions (Fig. 2f), we performed multiplexed T7-indexed chromatin immunoprecipitation (MINT-ChIP)^32^ on enhancer histone modification, H3K4me1, which also showed binding to this locus within W1. By contrast, accessibility did not occur until a week later (Fig. 3d). Immunofluorescence corroborated the delay in activating the *Runx1* locus, and underscored its prominence at later stages of reprogramming (Fig. 3e). Finally, footprint analyses exposed an increase in chromatin accessibility at RUNX footprints over the late-opening C4 ATAC peaks (Fig. 3f and Extended Data Fig. 3g). This contrasted with SOX footprints, which appeared early and then remained constant from W2 to W12 (Fig. 3f).

Gene Ontology (GO)-term analyses of the genes associated with these late-opening RUNX footprints reflected stem cell proliferation, and angiogenesis, hallmarks of cancers (Fig. 3g). Together, these data imply that later changes involved not only SOX9 but also transcription factors that were directly targeted by SOX9, notably of the RUNX family, whose motifs were also enriched as noted above. Although we did not address whether RUNX factors operate as pioneer factors, the enrichment of RUNX motifs coincident with the rise in proliferation during BCC-like downgrowth raised the possibility that proliferation may enhance if not allow accessibility of these factors to chromatin.

### SOX9 induces epigenetic remodelling before opening chromatin

**Fig. 4:**
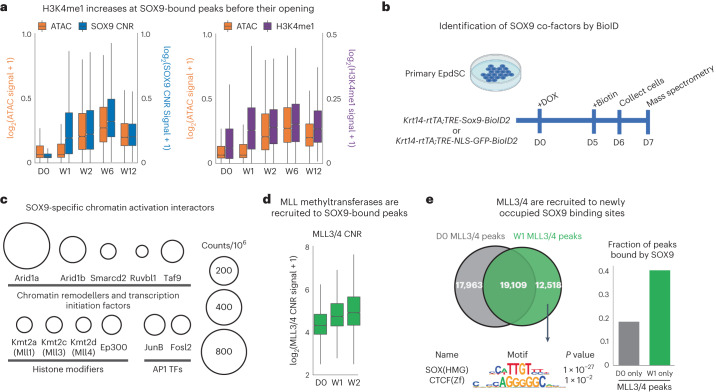
SOX9 recruits co-factors to epigenetically prime and then remodel chromatin to an open, transcriptionally accessible state. **a**, Left: box plot comparing ATAC (orange) and SOX9 CNR (blue) signals at SOX9 peaks that transition from closed to open chromatin over time. Note that SOX9 binding increases markedly by W1, preceding chromatin accessibility at these sites by nearly a week. Right: box plot comparing ATAC (orange) and MINT-ChIP H3K4me1 (purple) signals at SOX9 peaks that open over time. Note that H3K4me1 follows the time course of SOX9 binding, again preceding chromatin accessibility. *n* = 2 biological replicates. Box plots are centred at median and bound by first and the third quartile, and whiskers extend to 1.5 times interquartile range (IQR) on both ends. **b**, Schematic of BioID2 proximity labelling of proteins that interact with SOX9 induced in cultured EpdSCs. **c**, Selected SOX9-interacting proteins detected with mass spectrometry and that fall into the top GO terms of chromatin remodellers of the SWI/SNF family; transcription initiation factors (TAF9), AP1 transcription factors; and enzymes that modify histones (MLL1, MLL3, MLL4 and p300). For full list, see Supplementary Table 3. Circle size corresponds to the strength of the hit as delineated at right. **d**, Box plot of MLL3/4 CNR signals reveals the appearance of MLL3/4 at SOX9-bound peaks following DOX. *n* = 2 biological replicates. Box plot is centred at median and bound by the first and the third quartile, and whiskers extend to 1.5 times IQR on both ends. **e**, Venn diagrams providing further evidence that the de novo MLL3/4 peaks that appear between D0 and W1 are highly enriched for bound SOX9 (binomial *P* = 1 × 10^−27^), whereas the 17963 MLL3/4 peaks lost at W1 do not show any significant motif enrichment over all D0 MLL3/4 peaks and are not associated with SOX9-bound sites, inconsistent with a repressor role for SOX9.

**Extended Data Fig. 4:**
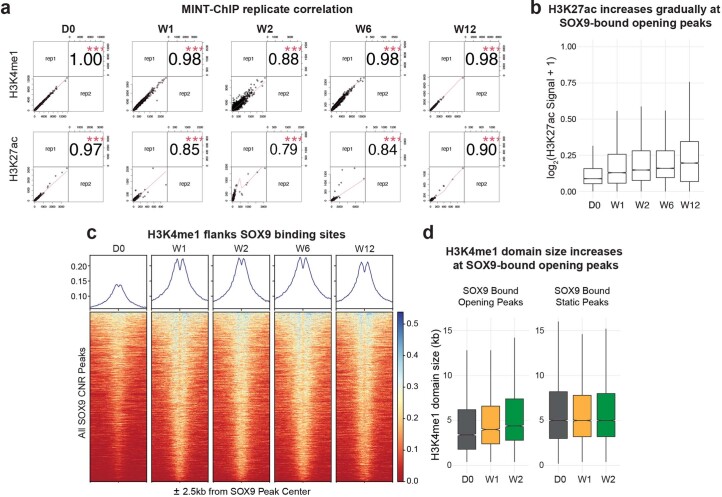
MINT-ChIP experiments and analyses of H3K4me1 relative to SOX9 binding. **a**, Replicate correlation analyses of the H3K4me1 and H3K27ac MINT-ChIP show strong correlation between each duplicate sample across all time points. **b**, Boxplot of H3K27ac signals at opening SOX9 peaks overtime. Note that the signal increases gradually from W1 to W12. n = 2 biological replicates. Boxplot is centered at median and bound by 1st and the 3rd quartile, and whiskers extend to 1.5 times IQR on both ends. **c**, Profile plot (top) and heatmap (bottom) showing the H3K4me1 signals at all SOX9 Cut-and-Run peaks across time points. Note the strong flanking pattern of the H3K4me1 signals adjacent to each SOX9 binding site (center dip) from W1 to W12. **d**, Boxplot of H3K4me1 domain sizes at opening SOX9 peaks and SOX9 bound static peaks shows gradual increase of H3K4me1 domain size from W1 to W2 after SOX9 induction. n = 2 biological replicates. Boxplots are centered at median and bound by 1st and the 3rd quartile, and whiskers extend to 1.5 times IQR on both ends.

The substantial delay between H3K4me1 and SOX9 versus chromatin accessibility and transcription of the *Runx1* gene led us to wonder whether this might be a general phenomenon of SOX9 reprogramming. To address this, we compared ATAC and histone modification signals over time at all opening SOX9 peaks (Fig. 2c). Correlating with SOX9 binding, H3K4me1 deposition occurred within W1 and levelled off thereafter (Fig. 4a), preceding chromatin accessibility changes at W2. By contrast, H3K27ac changes, while appearing by W1, were less robust and, in further contrast, continued to rise over time relative to SOX9 and H3K4me1 (Extended Data Fig. 4b).

Notably, although the nucleosomes directly over SOX9-binding sites appear to have been evicted, H3K4me1 was strongly enhanced on nucleosomes flanking SOX9 (Extended Data Fig. 4c). Moreover, the domain size of H3K4me1 gradually increased from D0 to W2. This did not occur at static peaks, but rather specifically at SOX9-bound opening peaks (Extended Data Fig. 4d).

### Activating HFSC enhancers

**Extended Data Fig. 5:**
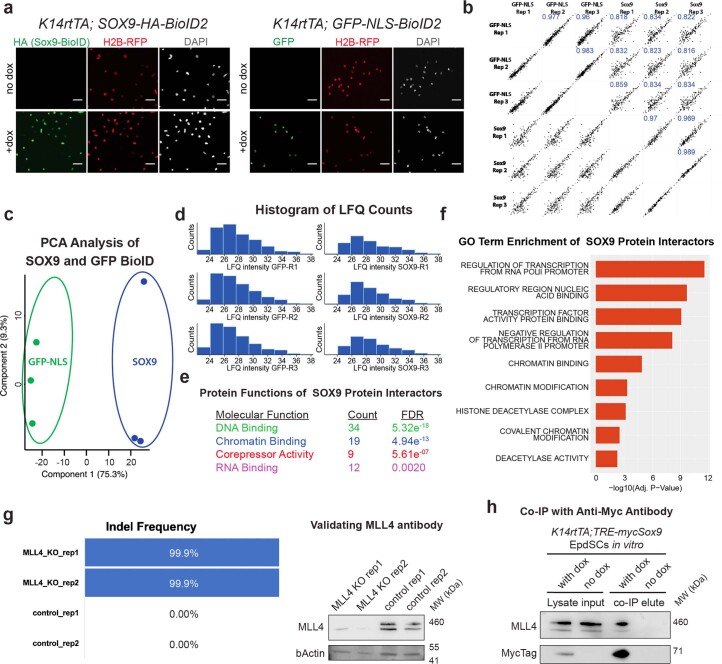
BioID experiments and identification of SOX9 co-factors. **a**, Immunofluorescence validation of *Krt14rtTA* primary keratinocytes transduced with TRE-HA-SOX9-BioID2; H2B-RFP (left) or TRE-GFP-NLS-BioID2; H2B-RFP (right). Transgenes were induced with doxycycline and immunolabeled for HA (left) or GFP (right). All scale bars are 50μm. **b**, Correlation plots of Label Free Quantification (LFQ) values identified by BioID experiments across replicates and samples. SOX9-BioID and GFP-NLS-BioID samples share stronger correlations between replicates than each other. Blue number represents r^2^ value. **c**, PCA analysis of GFP and SOX9 BioID protein interactors demonstrate sample-specific clusters. **d**, Histogram of LFQ intensity values for the GFP (left) and SOX (right) BioID replicates. **e**, Molecular function enrichment of proteins specifically interacting with SOX9. **f**, Gene ontology enrichment of proteins specific to SOX9 (binomial is used to calculate the significance). **g**, Two *Mll4* null keratinocyte cell lines (*Krt14rtTA; TRE-mycSox9*) were generated with CRIPSR/Cas9 with 99.9% indel frequency. These lines validated the efficacy of the MLL4 antibody by immunoblot, which detected a ~500 kDa protein in the control but not the knockout (KO) cell lines. MW, molecular weight. **h**, Immunoblot showing that the ~500 kDa protein, identified in (g) as MLL4, is pulled down with mycSOX9 in an anti-myc tag antibody immunoprecipitation. See also in associated source data.

To understand how SOX9 directly activates HFSC enhancers, we began by identifying SOX9-interacting co-factors. To this end, we transduced *Krt14-rtTA* primary EpdSCs in vitro with *TRE-Sox9-BioID2* and control *TRE-GFP-NLS-BioID2* and then induced expression of each transgene using DOX (Extended Data Fig. 5a). One week later, biotinylated SOX9-interacting proteins were purified and analysed by mass spectrometry (Fig. 4b).

Biological replicates correlated highly and formed distinct clusters by PCA (Extended Data Fig. 5b–d). Fifty-eight proteins interacted with SOX9 relative to NLS-GFP EpdSCs (Supplementary Table 3). On the basis of protein function and GO-term analysis, SOX9-interacting proteins were mainly DNA and chromatin binders enriched in chromatin modifications and nuclear activity. Among the strongest SOX9 interactions were with core members of the SWI/SNF chromatin remodelling complex (ARID1a/b and SMARCD2), TATA box binding protein TAF9 (TFIID) required for RNA polymerase II-mediated induction of transcription, and AP1 (FOSL2 and JUNB) (Fig. 4c and Extended Data Fig. 5e,f). Histone modifiers typifying key active enhancers in developmental contexts were also featured. As SOX9-induced opening peaks were more enriched at enhancers over promoters (Extended Data Fig. 3e), we were intrigued to find modifiers of two histone marks enriched at active enhancers: Ep300, the acetyltransferase for H3K27ac, and MLL3/MLL4, histone methyltransferases that not only can deposit H3K4me1 but possibly play additional emerging roles in enhancer activation^33–35^.

Since we observed an increase in H3K4me1 at SOX9 targeted enhancers before H3K27ac or chromatin opening, we first focused on whether, as predicted, MLL3/4 are recruited by SOX9 to closed chromatin in vivo. To validate the physical interaction, we exploited the MYC tag of SOX9 and performed co-immunoprecipitations on cultured EpdSC lysates with or without SOX9 induction, and then probed for MLL4. Given the large size of MLL4 (>500 kDa) and the likelihood of degradation, we used CRISPR–Cas9 to ablate *Mll4* in these EpdSCs to ensure correct band identification (Extended Data Fig. 5g).

After validation, we performed MLL3/4 CNR, reasoning that, if MLL3/4 recruitment to chromatin is regulated by SOX9, de novo MLL3/4 targeted sites should be enriched with SOX9 binding. A marked increase in MLL3/4 association with chromatin occurred between D0 and W2 at opening SOX9-bound enhancers (Fig. 4d). Moreover, upon analysing de novo MLL3/4 recruitment sites on chromatin at W1, we found that SOX motifs were significantly enriched (Fig. 4e). These data began to provide a clearer picture of how SOX9 functions as a pioneer factor, as it not only binds to closed chromatin but also recruits co-factors to epigenetically modify flanking histones. The data from Fig. 4c further hinted that SOX9 recruits the SWI/SNF complex to make the chromatin accessible for transcription.

### Silencing the epidermal fate

**Fig. 5:**
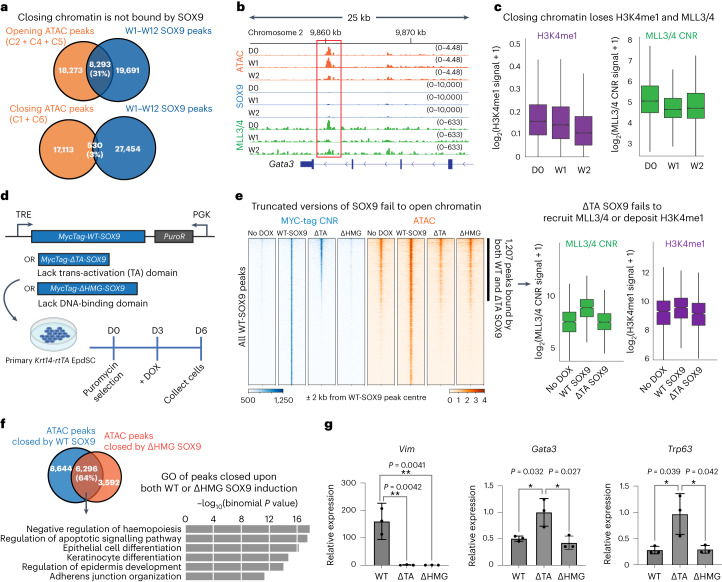
SOX9 achieves EpdSC fate silencing independent from DNA binding. **a**, Top: Venn diagram shows robust overlap between opening peak clusters (C2 + C4 + C5) and SOX9 peaks. Bottom: Venn diagram shows only 3% overlap between closing peaks (C1 + C6) and SOX9 peaks. **b**, ATAC, SOX9 CNR and MLL3/4 CNR tracks at the *Gata3* locus, showing that, by W1 after SOX9 induction, MLL3/4 CNR peaks were diminished, and by W2, ATAC peaks closed, even though CNR showed no SOX9 binding in this region (red box). **c**, Box plots showing loss of MLL3/4 and H3K4me1 signal beginning at W1 post SOX9-induction and specifically at ATAC peaks that close by W2 (C1, C6). *n* = 2 biological replicates. **d**, Schematic of inducing of MYC-tagged wild-type (WT) or mutant versions of SOX9 in transduced *Krt14-rtTA* cultured EpdSCs. **e**, Profile plot and heat map showing MYC-tagged wild-type or variant SOX9 binding (blue) and accessibility (orange) before and after DOX. Peaks are sorted the same way across samples. Note that ΔHMG-SOX9 fails to bind DNA, and ΔTA-SOX9 binds only to the subset of SOX9 peaks that were already accessible before DOX. Both mutants of SOX9 failed to open chromatin de novo. Right: box plot comparing MLL3/4 CNR and H3K4me1 signals at the peaks that are bound by wild-type SOX9 and ΔTA-SOX9. Note that only wild-type SOX9 brought additional MLL3/4 and deposited more H3K4me1 to these peaks. *n* = 2 biological replicates. **f**, Venn diagram shows overlap between the ATAC peaks that are closed by wild-type SOX9 or ΔHMG-SOX9 induction. GO terms reveal that epidermal enhancers close upon wild-type or ΔHMG-SOX9 induction (binomial is used to calculate the significance). **g**, Quantitative PCR analysis of genes that are directly induced (*Vim*) or indirectly repressed (*Gata3* and *Trp63*) by SOX9 in EpdSC cells. All the error bars are mean ± s.d. *P < 0.05 and **P < 0.01, two-tailed *t*-test. *n* = 3 biological replicates. All box plots are centred at median and bound by the first and third quartile, and whiskers extend to 1.5 times interquartile range (IQR) on both ends.

**Extended Data Fig. 6:**
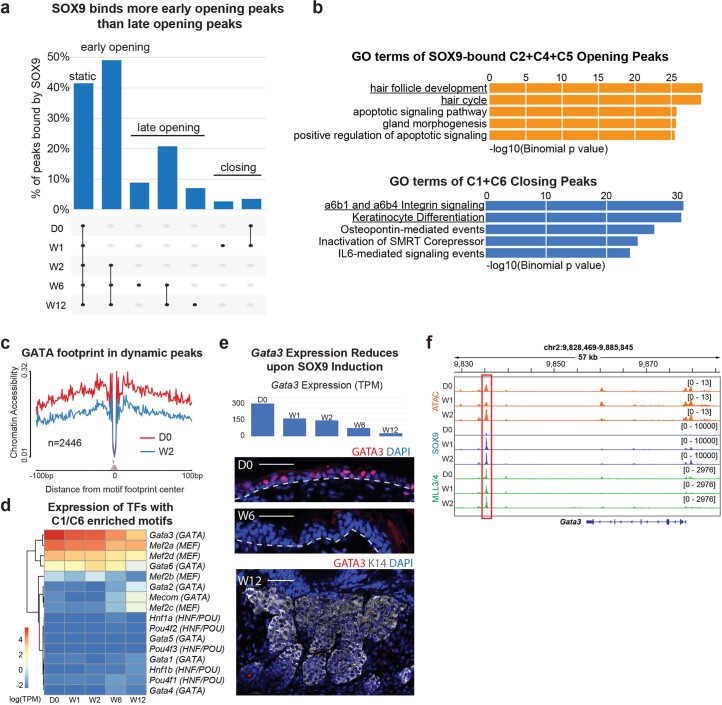
Epidermal TFs diminish rapidly upon SOX9 induction in EpdSCs. **a**, Upset plot shows % of peaks bound by SOX9 in the top peak sets from Fig. 2d. Note that peaks opened by W2 are more often bound by SOX9 than later opening or closing peaks. **b**, GO terms enriched in SOX9-bound opening peaks (C2, C4, C5) and all closing peaks (C1,C6). **c**, ATAC footprint analysis at D0 and W2 shows a decrease in chromatin accessibility at GATA footprint in dynamic ATAC peaks following SOX9 induction. **d**, EpdSCs expression of TFs that belong to TF families whose motifs are enriched in closing ATAC peaks (C1 and C6 in Fig. 3c) (binomial is used to calculate the significance). **e**, (top) Transcript levels of *Gata3* over time following SOX9 induction. (bottom) GATA3 immunofluorescence of epidermis at D0, W6 and W12. Scale bars, 50μm. **f**, Integrative Genomics Viewer (IGV) snapshot of SOX9, ATAC and H3K4me1 tracks within the *Gata3* locus. The red box indicates a peak >30kb downstream from the gene that is bound by SOX9 and MLL3/4, and stays open at W2 even though the gene body of *Gata3* closes its chromatin (see Fig. 5b).

Interestingly, while SOX9 binding was highly enriched within peaks that opened over time, it accounted for only 3% of peaks that closed over time (Fig. 5a). The differences were even more striking when we restricted our analysis to ATAC peaks changing over the first two weeks (Extended Data Fig. 6a). These findings were consistent with our SOX9 interactome, which was dominated by chromatin-activating remodellers. Moreover, in contrast to the hair-follicle-associated GO terms prominent in W2 SOX9-bound opening peaks, Epd-associated GO-terms were featured among closing peaks that were not bound by SOX9 (Extended Data Fig. 6b). We therefore hypothesized that SOX9 silences epidermal fate indirectly.

To further understand how epidermal fate is silenced, we were intrigued by GATA factors, whose motif was markedly enriched in ATAC peaks (C1, C6) that closed within the first 2 weeks after SOX9 induction and whose transcription factor footprint declined upon SOX9 induction (Extended Data Fig. 6c). GATAs surfaced upon analysing the transcription factors expressed by EpdSCs and whose motifs are highly enriched in closing chromatin (Extended Data Fig. 6d). GATA3 transcript and protein expression also declined concomitantly with the closure of GATA motifs (Extended Data Fig. 6e).

The *Gata3* gene locus also lost chromatin accessibility by W2, but the decline happened only at non-SOX9-bound peaks. The nearest SOX9-bound enhancer was >30 kb from the *Gata3* gene body, and like several other weaker peaks, this site was already open and MLL3/4-bound before SOX9 was induced. Notably, subsequent SOX9 binding had little or no effect on its status (Extended Data Fig. 6f). These findings suggest that the role of SOX9 in silencing epidermal fate is at least in part indirect. Moreover, the result appeared to be physiologically relevant as genes downregulated in SOX9^+^ embryonic skin progenitors were also silenced when SOX9 was induced in adult EpdSCs (Fig. 1d)^36^.

**Extended Data Fig. 7:**
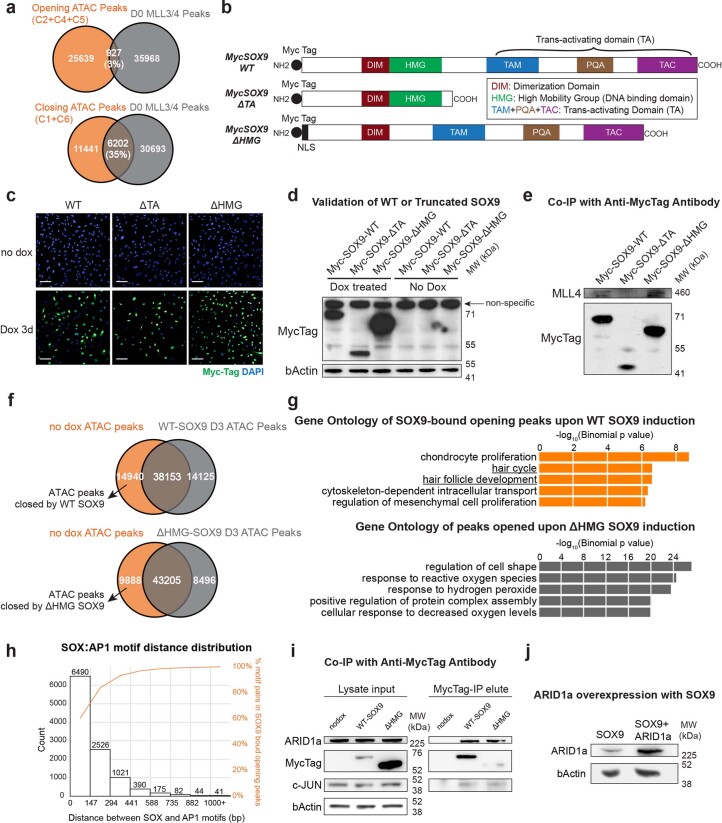
Truncated SOX9 displays impaired DNA binding or co-factor recruitment. **a**, (top) Venn diagram reveals almost no overlap between ATAC peaks (C2,C4,C5) that mostly open between W2-W12 and D0 MLL3/4 Cut-and-Run peaks. (bottom) Venn diagram shows substantial overlap between ATAC peaks (C1,C6) that mostly close between W1-W2 and D0 MLL3/4 Cut-and-Run peaks. **b**, Schematic illustrating constructs engineered to express WT SOX9 and two variants of SOX9. **c**, Immunofluorescence reveals similar intensities of WT and mutant SOX9 in induced EpdSCs. Scale bars, 100μm. **d**, Immunoblot validating the sizes of different versions of SOX9. MW, molecular weight. **e**, Immunoblot showing that the transactivating (TA) domain of SOX9 is sufficient in binding MLL4. **f**, Venn diagrams show that the peak sets closed by WT and ΔHMG-SOX9 are comparable in size. **g**, (top) Top 5 biological process gene ontologies of genes associated with SOX9-bound opening peaks upon WT SOX9 induction *in vitro* from GREAT. (bottom) Top 5 biological process gene ontologies of genes associated with peaks opened upon ΔHMG SOX9 induction *in vitro* from GREAT. Binomial is used to calculate the significance. **h**, Distribution of the distance between a SOX motif and its closest AP1 motif in the SOX9 bound opening peaks. Note that the x-axis is binned by multiplies of one nucleosome size (147bp). The cumulation plot of the distribution is shown in orange on the secondary y-axis. **i**, Immunoblot showing that both WT-SOX9 and ΔHMG-SOX9 are capable of binding c-JUN and ARID1a. **j**, Immunoblot validating ARID1a can be induced 3x higher in the SOX9 expressing keratinocytes. For full blots, see associated source data.

MLL3/4 presence over opening SOX9-dependent enhancer peaks was robust by W2, as was H3K4me1 modification (Fig. 4a,d). By contrast, the >6,000 SOX9-independent enhancer peaks that closed during this time displayed plummeting MLL3/4 association and a more gradual loss of H3K4me1 (Extended Data Fig. 7a). These findings raised the tantalizing possibility that, in binding to nucleosomes at HFSC-enhancers, SOX9 might be recruiting co-factors including MLL3/4 away from active EpdSC enhancers.

To test this hypothesis, we engineered DOX-inducible MYC-tagged wild-type and mutant forms of SOX9 that lacked either the transactivation (ΔTA) domain or the DNA binding (ΔHMG) domain (Fig. 5d and Extended Data Fig. 7b). In the transduced primary EpdSCs, immunofluorescence levels of three versions of SOX9 were comparable, and the ectopically expressed proteins were of the expected size (Extended Data Fig. 7c,d). Additionally, as judged by co-immunoprecipitation, only wild-type SOX9 and ΔHMG-SOX9, but not ΔTA-SOX9, associated with MLL4, consistent with the inability of ΔTA to interact with chromatin remodellers (Extended Data Fig. 7e).

By using CNR with a MYC-tag antibody recognizing all three SOX9 variants equivalently, we verified that wild-type SOX9 and the ΔTA-SOX9 mutant, but not ΔHMG-SOX9, bound to DNA (Fig. 5e). Interestingly, without the TA domain to interact with co-factors, ΔTA-SOX9 only bound to chromatin that was already accessible in EpdSCs. Consistent with this result, the 1,207 peaks that were open before DOX and bound by ΔTA-SOX9 did not show MLL3/4 recruitment nor did they show H3K4me1 modification (shown at right). Additionally, and in contrast to wild-type SOX9, ΔTA-SOX9 failed to stably bind to closed chromatin of HFSC enhancers, indicating that, without binding to co-factors, SOX9 lost the defining feature of pioneer factors.

Although ΔHMG-SOX9 did not bind DNA, it had a striking effect on chromatin accessibility. Nearly 10,000 ATAC peaks closed and >8,000 peaks opened upon induction (Extended Data Fig. 7f). As this mutant was unable to bind DNA, it was not surprising to see that the GO-term profile of the opening peaks was dramatically different than that of wild-type SOX9 (Extended Data Fig. 7g). Rather than HFSC features, the changes were more reflective of a stressed state. By contrast, in the ATAC peaks that closed in response to ΔHMG-SOX9, 64% of them were also closed by wild-type SOX9, and the GO terms corresponded to the same EpdSC genes indirectly silenced by wild-type SOX9 (Fig. 5f,g).

### Competition for SOX9-interacting chromatin remodellers

**Fig. 6:**
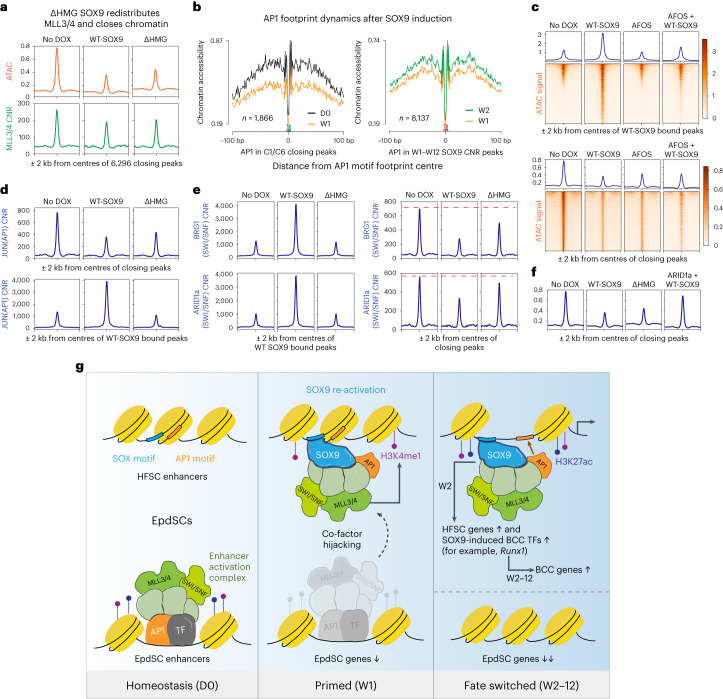
SOX9 redistributes chromatin remodelling co-factors to activate HFSC enhancers and silence EpdSC enhancers. **a**, Profile plots showing that ATAC and MLL3/4 CNR signals wane at EpdSC enhancers upon wild-type SOX9 and ΔHMG-SOX9 induction. Note that, when SOX9 cannot bind to DNA (ΔHMG), it still diminishes MLL3/4 at endogenous EpdSC enhancers and closes their chromatin. **b**, AP1 footprint analyses in closing ATAC peaks and SOX9 CNR peaks. Note that chromatin accessibility at AP1 footprints decreases over closing epidermal peaks by W1 and increases over opening SOX9-bound peaks by W2 (see also Fig. 5a). **c**, Top: profile plots and heat maps comparing ATAC signals at wild-type SOX9 (WT-SOX9) bound peaks in vitro. While WT-SOX9 can open chromatin, dominant negative FOS (AFOS) inhibits the opening as shown in the last column. Bottom: profile plots and heat maps comparing ATAC signals at closing peaks in vitro. AFOS phenocopies the indirect closing effect of SOX9 on AP1-associated epidermal enhancers. **d**, Top: profile plots showing that JUN CNR signals are reduced at EpdSC enhancers upon wild-type SOX9 and ΔHMG-SOX9 induction. Bottom: supportive of competition, wild-type SOX9 recruits AP1 to HFSC enhancers, while ΔHMG-SOX9, lacking the DNA binding domain, fails to do so. **e**, Profile plots showing that both BRG1 and ARID1a of the SWI/SNF complex behave similarly to AP1 and MLL3/4: they are recruited to the opening HFSC enhancers by only wild-type SOX9 (left), while both wild-type SOX9 and ΔHMG-SOX9 reduce SWI/SNF association with epidermal enhancers (right). The red dotted line denotes CNR levels of indicated target at closing peaks before DOX. **f**, Profile plots comparing ATAC signals at closing epidermal enhancers with wild-type SOX9, ΔHMG-SOX9 or ARID1a overexpression together with wild-type SOX9. Note that, when ARID1a is overexpressed, SOX9 fails to close epidermal enhancers. **g**, Working model for how SOX9 achieves cell fate switching: pioneer factor SOX9 indirectly silences the epidermal fate by competing away co-factors and other transcription factor (TFs) including AP1 from active EpdSC enhancers. Concomitantly, SOX9 binds directly to key hair follicle enhancers, bringing with it the hijacked chromatin remodelling machinery and activating the hair follicle fate. Also among the SOX9 target genes are transcription factors such as RUNX1, whose footprints appear to participate in the delayed activation of BCC cancer genes, leading to further fate switching to BCC, downstream of the EpdSC-to-HFSC fate transition.

Consistent with the hypothesis that SOX9 closes chromatin by competing for and redistributing co-factors, MLL3/4 CNR signal diminished over EpdSC enhancers upon ΔHMG-SOX9 induction (Fig. 6a). Probing deeper, we turned to AP1 transcription factors, which surfaced in our SOX9 interactome. In agreement with the dynamics observed for MLL3/4, footprint analysis in vivo revealed that AP1 binding decreased in closing non-SOX9-bound epidermal enhancers and increased in SOX9-bound chromatin (Fig. 6b). Moreover, in these SOX9-bound opening peaks, SOX and AP1 motifs were mostly found within one-nucleosome distance, supporting a role for SOX9 in targeting AP1 transcription factors to their canonical binding sites upon opening hair follicle enhancers (Extended Data Fig. 7h).

Notably, motif analyses revealed the presence of AP1-binding sites in both closing and opening enhancers (Extended Data Fig. 3f), suggesting that the interaction between SOX9 and AP1 may be functionally important for both opening SOX9^+^ HFSC enhancers and closing SOX9^neg^ EpdSC enhancers. To test the possibility that enhancers might be competing for AP1 binding, we used the strategy delineated in Fig. 5d to induce AFOS, a dominant negative version of c-FOS that can heterodimerize with AP1 transcription factors and block their binding to DNA^37,38^. We performed these experiments in the presence and absence of wild-type SOX9, and then carried out ATAC-seq. In the peaks that were bound by wild-type SOX9, AFOS clearly interfered with the opening of the HFSC enhancers, while also phenocopying the closing effects of SOX9 at EpdSC enhancers when expressed alone (Fig. 6c).

Our data thus far suggested that, like MLL3/4, AP1 transcription factors function on both sides of the fate coin. To test whether other members of the interactome are targets for this putative competition for SOX9-binding partners, we focused on AP1 transcription factors and the SWI/SNF complex. After first validating their association with SOX9 (Extended Data Fig. 7i,j), we performed CNR analysis. We found that induction of wild-type SOX9 resulted in increased JUN(AP1) binding at SOX9-bound peaks, and decreased JUN binding at closing epidermal peaks. Notably, while ΔHMG-SOX9 failed to recruit JUN and open hair follicle enhancers, it still diminished JUN binding at closing epidermal peaks (Fig. 6d). Similarly, when we performed CNR on both structural (ARID1a) and enzymatic (BRG1) members of the SWI/SNF complex, we observed a decline in their association with epidermal enhancers when either wild-type SOX9 or ΔHMG-SOX9 were induced, but an increased association with SOX9-bound peaks only after wild-type SOX9 and not ΔHMG-SOX9 induction (Fig. 6e).

Together, these data suggest that SOX9 orchestrates the redistribution of transcription factors and epigenetic co-factors that are shared by the enhancers of both cell fates. Moreover, this competition appeared to be predicated in part on limiting levels of chromatin remodelling factors, as when we overexpressed ARID1a in the presence of SOX9, epidermal enhancers were rescued from closing (Fig. 6f and Extended Data Fig. 7i,j).

## Discussion

Elegant studies by the Zaret lab launched the field of pioneer factors, now examined in various fate-switching scenarios and distinguished by their ability to bind their sequence motifs within closed chromatin^1,2^. However, the precise sequence of nucleosome eviction, opening of surrounding chromatin, and reprogramming fate choices has been difficult to unravel, particularly in in vitro settings, where fate choices lack constraints imposed by native tissue microenvironments. By exploiting the slowed kinetics of our in vivo reprogramming system, we discovered that SOX9 not only perturbs its target nucleosome but also recruits enzymes that modify the flanking enhancer nucleosomes. Like SOX9 binding itself, these features precede the subsequent chromatin opening to the transcriptional machinery. As our ΔTA-SOX9 studies imply, these dynamics appear to be achieved by SOX9 recruiting of chromatin remodelling factors such as AP1 TFs and the SWI/SNF complex^39,40^.

It has generally been viewed that a pioneer factor can act either as a transcriptional activator or as a repressor through recruiting different cohorts of co-activators or co-repressors^1,2^. At first glance, this notion seems well suited to nodes of lineage switching, where one fate is silenced while another is chosen. However, increasing evidence suggests that pioneer factors may bind and directly regulate the enhancers of only one lineage at the crossroads, leaving a conundrum as to how the other lineage becomes silenced to achieve the switch.

Our findings showed clearly that EpdSC gene silencing occurs shortly after SOX9 induction, a timing that is at odds with the notion that SOX9 might induce transcriptional repressors that then subsequently silence epidermal genes. Moreover, in contrast to HFSC enhancers, many of which bind SOX9 and are opened de novo, EpdSC enhancers show a paucity of SOX9 binding and yet close rapidly upon SOX9 induction.

Rather to prevailing notions, our findings favour a dual function model whereby a pioneer factor actively hijacks and redistributes shared co-factors to achieve cost-effective and coordinated fate switching from one lineage to another (Fig. 6g). Thus, following SOX9 induction in EpdSCs, MLL3/4 binding increased at SOX9-bound opening HFSC enhancers, while diminished at closing non-SOX9-bound EpdSC enhancers. Our studies with wild-type SOX9 and ΔHMG-SOX9 revealed that not only does SOX9 interact with MLL3/4, but also with a compendium of co-factors essential to activate enhancers, which include not only MLL3/4 but also AP1 and SWI/SNF complex.

In closing, although direct repressive mechanisms independent from chromatin accessibility are still formally possible, our data suggest that at least some chromatin remodellers that are generally required for enhancer activity are in short supply, thereby setting up the competition to achieve fate switching once a pioneer factor such as SOX9 is activated. By utilizing such a mechanism, cellular fate plasticity is minimized, while simultaneously expediting the shift in density of shared transcriptional regulators to genomic loci of new fate determinants.

Finally, it is noteworthy that, to make tissue, stem cells must undergo a fate choice, which for SOX9^+^HFSCs, is achieved by downregulating SOX9 (ref. ^16^). In our model as in BCC, SOX9 was constitutive and hence the choice to make hair was never made. Moreover, when left outside the instructive microenvironment of the quiescent hair follicle bulge niche, the proliferating cells with sustained SOX9 activated SOX9 downstream target transcription factor genes, such as those encoding the RUNX family, that secondarily drove further dynamic changes in the chromatin landscape. These findings begin to explain how and why in adult tissue stem cells, sustained re-activation of a pioneer factor involved in embryonic fate decisions frequently leads to cancer^5,6,41^.

## Methods

### Ethical regulation compliance

All animals used in this study were maintained and bred under specific-pathogen-free conditions at the Comparative Bioscience Center at The Rockefeller University, which is an Association for Assessment and Accreditation of Laboratory Animal Care-accredited facility. All procedures were performed with the Institutional Animal Care and Use Committee-approved protocols (20012-H and 20066-H).

### Generating and handling *TRE-Sox9* mice

To generate the conditional SOX9 transgenic mice, the *Sox9* coding sequence was cloned into the pTRE2 vector harbouring a DOX-inducible, minimal CMV2 promoter. A MYC tag was added to the N terminus of SOX9. Transgenic mice were generated as described previously^42^. The resulting *TRE-Sox9* mice were then genotyped and crossed to *Krt14-rtTA* transgenic mice^15^ to allow for DOX-inducible expression of MYC–SOX9 specifically in skin epithelium.

### Primary cell isolation

Primary *Krt14-rtTA*;*TRE-Sox9* EpdSCs were isolated from newborn male pups (postnatal day 0, or P0) as described previously^16,43^. Briefly, mouse back skin was collected from P0 pups and treated with dispase (Gibco) overnight at 4 °C. Epidermis was manually separated from dermis and disassociated into a single-cell suspension. Epidermal cells were passaged and maintained in E-low calcium medium^44^ (0.05 mM CaCl_2_) at 37 °C with 7.5% CO_2_.

### DOX treatment

A total of 0.1 mg of DOX (Sigma) in 100 μl phosphate-buffered saline (PBS) was administered by intraperitoneal injection to *Krt14-rtTA*;*TRE-Sox9* and *Krt14-rtTA*-only mice at postnatal day P21, and the mice were thereafter were maintained on mouse chow containing 2 mg g^−1^ DOX throughout the experimental time course. Phenotypic mice were housed with at least one control littermate for adequate grooming. To maintain proper body fluid, 100 μl PBS was administered through intraperitoneal injection every other day after 4 weeks of SOX9 induction. For W12 samples, epidermis from the back skin of P0 *Krt14-rtTA*;*TRE-Sox9* or *Krt14-rtTA*-only pups were grafted onto 6–8-week-old immunocompromised (*Nude*) female mice. Grafts were allowed to heal for 21 days, and DOX was administered as above. For induction of SOX9 and its variants, AFOS and ARID1a in cultured cells, DOX was added to a final concentration of 1 μg ml^−1^ in E-low medium for BioID or SOX9 variant experiments.

### Immunofluorescence

Mouse back skin was fixed in 4% paraformaldehyde at room temperature for 15 min, and then washed three times with PBS for 15 min at 4 °C. Following PBS washes, samples were dehydrated in 30% sucrose in PBS 4 °C overnight. The dehydrated samples were then embedded in optimal cutting temperature (OCT) medium (VWR) and frozen on dry ice. Cryosections (16 μm) were blocked in immunofluorescence buffer containing 0.3% Triton X-100, 2.5% normal donkey serum, 2.5% normal goat serum, 1% bovine serum albumin and 1% gelatin in PBS for 1 h at room temperature. After blocking, the sections were stained with primary antibodies in immunofluorescence buffer at 4 °C overnight: MYC-tag (rabbit, 1:1,000, Cell Signaling), SOX9 (rabbit, 1:5,000, Millipore), ITGA6 (rat, 1:1,000, BD), KRT14 (chicken, 1:1,000, BioLegend), KRT10 (rabbit, 1:250, Fuchs Lab), EpCAM (rabbit, 1:100, Abcam), KRT6 (guinea pig, 1:1,000, Fuchs Lab), RUNX1 (rabbit, 1:100, Abcam), and GATA3 (rat, 1:100, Invitrogen). After primary antibody staining, all sections were washed three times with immunofluorescence buffer containing 0.1% Triton X-100 in PBS for 5 min at room temperature. Sections were then stained with Alexa 488, 546 or 647 conjugated secondary donkey antibodies (1:500, Thermo Fisher), mounted with Prolong Diamond anti-fade mounting medium with 4′,6-diamidino-2-phenylindole (DAPI, Thermo Fisher) and imaged with Zeiss Axio Observer Z1 with Apotome 2 microscope. Images were collected and analysed with Fiji (ImageJ v.2.3.0). For the Human Atlas immunostaining, the following antibodies were used: SOX9 (CAB068240), EpCAM (CAB030012) and KRT6A (HPA061168).

For cultured cells, cells were plated onto chamber slides (Thermo Fisher). At collection, cells were fixed with 4% paraformaldehyde for 10 min, and then washed three times with PBS at room temperature. After washing, the cells were blocked and stained with primary antibodies the same way as described above for sections with the following primary antibodies: HA-tag (rabbit, 1:1,000, Cell Signaling), GFP (chicken, 1:2,000, Fuchs Lab), RFP (rat, 1:1,000, ChromoTek), and MYC-tag (rabbit, 1:1,000, Cell Signaling).

For 5′-ethynyl-2′ deoxyuridine (EdU) experiments, mice were injected IP with EdU (50 μg g^−1^ body weight) 2 h before analysis. Quantifications were performed by counting the number of EdU^+^ EpdSCs within the basal layer. For quantifying the SOX9 signal in the native ORS and the SOX9-induced epidermis, sections were stained with same SOX9 antibody concentration (1:5,000), and same laser intensity and exposure time were used to acquire images. From each sample, 100 cells were quantified with the multi-point tool in Fiji.

### Flow cytometry and cell sorting

*Krt14-rtTA*;*TRE-Sox9* and *Krt14-rtTA*-only male mice were used for FACS experiments to obtain maximal cell numbers and to control for variation due to sex. Briefly, the whole back skins were first dissected from the mouse. After scraping off the fat tissues from the dermal side, the tissues were incubated in 0.25% trypsin/ethylenediaminetetraacetic acid (EDTA) (Gibco) for 45–60 min at 37 °C. After quenching the trypsin with cold FACS buffer (5% foetal bovine serum, 10 mM EDTA and 1 mM HEPES in PBS), the epidermal layer and HFs were scraped off the epidermal side of the skin. The tissues were mechanically separated and filtered through a 70 μm cell strainer (BD) into a single-cell suspension for immunolabelling. Single-cell suspensions were immunolabelled with antibodies: Ly6A/E-APCCy7 (1:500, BioLegend), CD49f-PECy7 1:1,000, BioLegend), CD34-Alexa660 (1:50 Invitrogen), CD45-biotin (1:200, BioLegend), CD31-biotin (1:200, BioLegend), CD140a-biotin (1:200, BioLegend), CD117-biotin (1:200, BioLegend), TruStain FcX for blocking (1:1,000, BioLegend) and streptavidin-FITC (1:1,000, BioLegend) in 300 μl of FACS buffer. Stained cells were washed and resuspended with FACS buffer with 100 ng ml^−1^ DAPI before analysis or sorting. EpdSCs were collected using an Aria Cell Sorters (BD Biosciences) with BD FACSDiva (v. 8.0) into either FACS buffer for genomic experiments or TRIzol LS (Invitrogen) for RNA extraction.

### RNA-seq and raw file processing

EpdSCs were collected by FACS as described above directly into TRIzol LS (Invitrogen). RNA libraries were generated using SMARTer RNA kit for low-input RNA-seq. Libraries were sequenced on Illumina NovaSeq SP. Raw FASTQ files were trimmed of barcodes using Skewer (v.0.2.2) and transcript abundance quantified using Salmon (v.1.4.0) with a modified GENCODE transcript index (version GRCm38 release M24) to include *TRE-Sox9*. Gene level counts and transcripts per million (TPM) were calculated using the Tximport (v.1.12.3) package in R (v.3.6.1). For hair placode RNA-seq data, after generating the raw counts, differentially expressed (DEG) gene list was generated with DESeq2 (v.1.16.1).

### ATAC-seq and raw file processing

ATAC-seq^20^ was performed on FACS-purified EpdSCs (two to four male mice per replicate) at indicated timepoints (D0, W1, W2, W6 and W12) and cultured keratinocytes. Briefly, cells were lysed in ATAC lysis buffer for 5 min and then transposed with Tn5 transposase (Illumina) for 30 min. Samples were barcoded and sequencing libraries were prepared according to the manufacturer’s guidelines (Illumina) and sequenced on an Illumina NextSeq. For sequencing analysis, 50 bp paired-end FASTQs were aligned to the mouse genome (GRCm38/mm10) using the PEPATAC (v0.10.3) pipeline^45^. Replicate BAM files were merged, and peak calling was performed using Model-based Analysis of ChIP-Seq 2 (MACS2) with the option of ‘–keep-dup all’ to keep duplicates generated during the combining of experimental replicates. Because peak calling is greatly influenced by number of reads and sequencing depth, we normalized peak calling as performed as described^21^ with a threshold of 3, and we quantified reads in filtered peaks (RIP) for generating normalized bigwig files. To do so, 1,000,000/RIP was used as input for Deeptools ‘bamcoverage’ with the ‘–scaleFactor’ option. Shared peaks were defined as regions that had ≥1 base pair overlap between two timepoints as shown in Fig. 2e. Dynamic peaks were defined as those accessible chromatin regions that were absent from at least one timepoint. For PCA analysis, peaks called from combined replicates were merged to create a union set of peaks across the samples. Read counts under the union peaks were summed for each individual replicate and used as input for PCA analysis or generating *K*-means clusters in R.

### CNR and raw file processing

EpdSCs were FACS purified, and the CNR sequencing was performed as previously described^19,46^ with minor modifications indicated below. Briefly, 500,000–1,000,000 EpdSCs were washed with ice-cold PBS, resuspended in crosslinking buffer (10 mM HEPES–NaOH pH 7.5, 100 mM NaCl, 1 mM egtazic acid (EGTA), 1 mM EDTA and 1% formaldehyde) and rotated at room temperature for 10 min. Crosslinked cells were quenched with glycine at a final concentration of 0.125 M for 5 min at room temperature. Cells were washed with cold 1× PBS and resuspended in NE1 buffer (20 mM HEPES–KOH pH 7.9, 10 mM KCl, 1 mM MgCl_2_, 1 mM dithiothreitol, 0.1% Triton X-100 supplemented with Roche complete protease inhibitor EDTA-free) and rotated for 10 min at 4 °C. Nuclei were washed twice with CNR wash buffer (20 mM HEPES pH 7.5, 150 mM NaCl, 0.5% bovine serum albumin and 0.5 mM spermidine supplemented with protease inhibitor) and incubated with concanavalin-A (ConA) beads washed with CNR binding buffer (20 mM HEPES–KOH pH 7.9, 10 mM KCl, 1 mM CaCl_2_ and 1 mM MnCl_2_) for 10 min at 4 °C. ConA-bead-bound nuclei were incubated overnight at 4 °C in CNR antibody buffer (CNR wash buffer supplemented with 0.1% Triton X-100 and 2 mM EDTA) and antibody. After antibody incubation, ConA-bead-bound nuclei were washed once with CNR Triton wash buffer (CUT&RUN wash buffer supplemented with 0.1% Triton X-100) then resuspended and incubated at 4 °C for 1 h in CUT&RUN antibody buffer and 2.5 μl pAG-MNase (EpiCypher). ConA-bound-nuclei were then washed twice with CUT&RUN Triton wash buffer and resuspended in 100 μl of Triton wash buffer and incubated on ice for 5 min. Then, 2 μl 100 mM CaCl_2_ was added and mixed gently to each 100 µl ConA-bound-nuclei. The reaction was then incubated at 0 °C for 30 min. The reaction was stopped by addition of 100 μl 2× stop buffer (340 mM NaCl, 20 mM EDTA, 4 mM egtazic acid, 0.1% Triton X-100 and 50 μg ml^−1^ RNaseA) and incubated at 37 °C for 10 min. All buffers mentioned above were filtered with 0.22 μm filter before use. After incubation, ConA-bound-nuclei were captured using a magnet and supernatant containing CNR DNA fragments were collected. Supernatant was incubated at 70 °C for 4 h with 2 μl 10% sodium dodecyl sulfate and 2.5 μL 20 mg ml^−1^ proteinase K. DNA was purified using PCI reagent (phenol:chloroform:isoamyl alcohol, Millipore) and overnight ethanol precipitation with glycogen at −20 °C. DNA was resuspended in elution buffer (1 mM Tris–HCl pH 8.0 and 0.1 mM EDTA).

CNR sequencing libraries were generated using NEBNext Ultra II DNA Library Prep Kit for Illumina and NEBNext Multiplex Oligos for Illumina. PCR-amplified libraries were purified using 1× ratio of SPRI beads (Beckman) and eluted in 15 μl EB buffer (Qiagen). All CNR libraries were sequenced on Illumina NextSeq using 40 bp paired-end reads. Reads were trimmed with Skewer and aligned to reference genome (mm10) using Bowtie2 (v.2.2.9) and deduplicated with Java (v.2.3.0) Picard tools (http://broadinstitute.github.io/picard). Reads were filtered to ≤120 bp using Samtools (v.1.3.1). BAM files for each replicate were combined using Samtools. Bigwig files were generated using Deeptools (v.3.1.2) with reads per kilobase of transcript per million mapped reads (RPKM) normalization and presented with Integrative Genomics Viewer software. CNR peaks were called using SEACR^47^ from bedGraph files generated from RPKM-normalized Bigwig files (bigWigToBedGraph, UCSC Tools) using stringent setting and a numeric threshold of 0.01. Peaks were further filtered to have peaks scores >1,800 for a set of high-confidence peaks.

### MINT-ChIP–seq and raw file processing

EpdSCs were FACS purified and subjected to histone ChIP–seq (MINT-ChIP) with antibodies recognizing H3K4me1 (rabbit, Cell Signaling), H3K27ac (rabbit, Active Motif) and Total H3 (mouse, Active Motif). Pooled samples were then sequenced using 50 bp paired-end Illumina NextSeq. Resulting FASTQ files were demultiplexed for specific histone antibodies by using the unique barcode present in sequenced read2. Resulting paired reads were then trimmed for adapters using Skewer and aligned to mouse genome (GRCm38/mm10) using Bowtie2. Duplicated reads were marked and removed using Picard, and replicates were merged with Samtools. Peak calling for H3K27ac was performed using MACS2, while broad domains of H3K4me1 were called using epic2 (ref. ^48^). Samples were independently normalized to the number of RIP. For visualization, Bigwig files were generated on the combined BAM files using Deeptools ‘bamcoverage’ with (1,000,000/RIP) as input for the ‘–scalefactor’ option. For total H3, RPKM was used for normalization.

### BioID and mass spectrometry

For identification of SOX9-interacting partners we transduced primary *Krt14-rtTA* EpdSCs with LV-TRE-MYC-BioID2-GFP-NLS-H2B-RFP or LV-TRE-MYC-BioID2-SOX9-H2B-RFP. RFP^+^ transduced cells were then isolated using FACS, and stable EpdSC lines were established. We induced expression of recombinant proteins using 1 μg ml^−1^ DOX. Cells were allowed to expand for 5 days and were pulsed with 50 μM biotin (Sigma) for 16 h before reaching confluence. Cells were purified and proteins isolated as previously described^49^ with minor modifications mentioned below. Immediately after sonication, lysates were washed using Zeba desalting columns (7K molecular weight cut-off, ThermoFisher cat. no. 89894) with 50 mM Tris pH 7.4 to remove excess biotin. Beads were also washed three times with 2 M urea and a final two times with PBS before being resuspended with 500 μl 50 mM Tris, pH 8.0. All washes were performed using a magnetic stand. New tubes were used in between each urea and PBS washes. Wash buffer was removed from suspension of magnetic beads and replaced with 100 μl 8 M urea, 50 mM ammonium bicarbonate and 10 mM dithiothreitol for 1 h and replaced with 100 μl 40 mM iodoacetamide and incubated in the dark for 30 min. Alkylation solution was replaced with 1 μg trypsin (Promega) dissolved in 100 μl 50 mM ammonium bicarbonate and incubated for 4 h. Supernatant was then removed and re-digested overnight using 0.5 μg trypsin and 0.5 μg Endopeptidase Lys-C (Wako). Peptides were desalted and concentrated using C18-based Stage tips^50^ and separated by nanoLC (gradient: 2% B/98% A to 38% B/62% A in 70 min, A: 0.1% formic acid, B: 90% acetonitrile/0.1% formic acid) coupled to a Fusion Lumos (Thermo Scientific) operated in high/high mode.

Data were queried with UniProts Complete Proteome mouse database and concatenated with known common contaminants. Proteome Discover and Mascot was used to analyse the resulting data produced. Data were further filtered using a percolator^51^ to calculate peptide false discovery rates and set a threshold of 1%. Proteins were specific to SOX9’s proximity if they were identified in two of the three MYC-BioID2-SOX9 replicates and absent from all the MYC-BioID2-GFP-NLS samples. For the full list of SOX9-specific interactors and raw counts, see Supplementary Table 3.

### Generation of EpdSC lines expressing SOX9 and variants, AFOS or ARID1a

Three versions of MYC-tagged SOX9 (WT, ΔTA and ΔHMG as indicated in Extended Data Fig. 7b) were cloned into plKO vectors with a TRE promoter and a puromycin-resistance gene (*puroR*) under the control of a constitutive promoter (PGK). Three lentiviruses were produced as described^52^. *Krt14-rtTA* EpdSCs were cultured and transduced with 1 μl concentrated lentivirus in 10 ml E-low medium with 8 μg ml^−1^ polybrene (hexadimethrine bromide, Sigma 107689-100MG) overnight. Transduced cells were then selected with 2 μg ml^−1^ puromycin for 5 days before DOX treatment. For AFOS and ARID1a experiments, Flag-tagged *AFOS* or *Arid1a* CDS were cloned into the described plKO vector for lentiviral production. *Krt14-rtTA* or *Krt14-rtTA;TRE-mycSOX9* EpdSCs were cultured and transduced with 1 μl concentrated lentivirus as described above. Transduced cells were also selected with puromycin for 5 days before DOX treatment.

### CRISPR-mediated *Mll4* knockout

To generate *Mll4* (also known as *Kmt2d*) null lines, we cultured keratinocytes from the EpdSCs of our *Krt14-rtTA, TRE-Sox9* mice. Lines were generated with the Alt-R CRISPR–Cas9 system (Integrated DNA Technologies). Briefly, a recombinant Cas9 protein, a validated single guide RNA (TGCTCGGCAACAGACGTGAC) targeting *Mll4* or a negative control single guide RNA (Integrated DNA Technologies), and an ATTO-550 conjugated tracer RNA were used to form a ribonucleoprotein were mixed with RNAiMax reagent (Thermo Fisher). Then, keratinocytes were transfected with the mixture overnight, and FACS purified into 96-well plates to produce clonal cell lines. The knockout cell lines were validated through sequencing of the target region for indel efficiency via MiSeq and used for the immunoblot of MLL4.

### Immunoblotting and co-immunoprecipitation

Cultured EpdSCs were washed on the plate in cold 1× PBS, lysed in RIPA buffer (Millipore) supplemented with protease and phosphatase inhibitors (Roche), and collected by scraping. Cells were lysed for 15 min on ice and then centrifuged to collect the supernatant. Co-immunoprecipitation was performed as previously described^53^ with the modification where protein-A/G-conjugated magnetic beads (Pierce) were used to bind antibodies instead, and proteins were eluted from beads with 1× NuPAGE LDS Sample Buffer (Invitrogen) with 2.5% 2-mercaptoethanol at 70 °C for 10 min. Protein concentration was determined by BCA Assay (Pierce) against a bovine serum albumin standard curve. Then 15 μg protein of each sample was run on NuPAGE 4–12% Bis-Tris Gels (Invitrogen) for 2 h at 110 V in NuPAGE MOPS SDS Running Buffer (Invitrogen). Protein was transferred onto nitrocellulose membrane (Cytiva) in NuPAGE Transfer Buffer (Invitrogen) at 15 V overnight at 4 °C. Given the marked differences in expected sizes of some of the proteins, overlapping host species of the antibodies raised, and the paucity of primary cell lysates for immunoprecipitates, we often cut the blots on the basis of size and performed immunoblotting on each piece with different antibodies. Membranes were then treated with blocking buffer with 5% non-fat dry milk and 0.1% Tween-20 in TBS for 1 h at room temperature before incubating with primary antibodies. The following primary antibodies were diluted in blocking buffer: MYC-tag (mouse, 1:1,000, Cell Signaling), MLL4 (mouse, 1:200, Santa Cruz Biotechnology), cJUN (rabbit, 1:1,000, Cell Signaling), ARID1a (rabbit, 1:1,000, Abcam) and β-actin (mouse, 1:10,000, Cell Signaling). The membranes were incubated in primary antibodies overnight at 4 °C. Membranes were then washed three times in 0.1% Tween-20 in TBS before incubating with HRP secondary (1:10,000) antibody for 1 h at room temperature. After secondary antibody incubation, membranes were then washed four times in 0.1% Tween-20 in TBS and incubated in ECL Prime reagents (Cytiva) for 5 min before chemiluminescence detection. Membranes were imaged with an GE Amsham AI600 Imager. For clarity, we show the bands of the correct sizes. However, all full blots (cut before processing as delineated above) are shown in corresponding source data.

### Quantitative PCR

Equal amounts of RNA extracted from cultured cells were collected with AllPrep DNA/RNA Kits (Qiagen) and reverse transcribed using the superscript VILO cDNA synthesis kit (Invitrogen). For quantitative PCR, biological replicates represent the average of three technical replicates per individual sample. Complementary DNAs from each sample were normalized using primers against *Rps16*. All primers used are provided in Supplementary Table 4.

### Bioinformatic analyses

#### GSEA

For comparing with both hair placodes and BCC, TPM matrices in D0, W2 and W12 were used as GSEA (v. 4.1.0) input. The DEG lists as illustrated in Fig. 1d were used as gene set inputs. For the BCC sample, DEG list of genes with *P* < 0.05 was curated from GSE152487 in the Gene Expression Omnibus depository^17^. GSEA was run with default settings, without collapsing, and with the gene set as the permutation type. The leading-edge analysis function was used to determine the significance of gene set enrichment.

#### Heat maps and box plots

All heat maps showing sequencing signals over binding sites are generated with Deeptools from RIP- or RPKM-normalized bigwig files. Profileplyr (v. 1.4.3) was used to generate ATAC, H3K4me1, H3K27ac and MLL3/4 CNR box plots in R with matrix output from Deeptools compute-matrix as input. The histone H3 profile plot was also generated with Profileplyr in R.

#### GO analysis

We performed GO analysis of each ATAC-seq cluster by associating each region with genes and performing enrichment analysis using Genomic Regions Enrichment of Annotation Tool (GREAT, version 3)^54^ with default gene association settings and the whole mouse genome (GRCm38/mm10) as the background.

#### Transcription factor motif and footprint analyses

For motif enrichment analysis on peak sets, HOMER^55^ (v. 4.10) findMotifGenome.pl was used with a customized motif database from JASPAR2018 (ref. ^56^). The motif input for HOMER was generated from the 79 clusters of JASPAR2018 vertebrates CORE central transcription factor motifs using 80% of the maximum log-odds expectation for each motif as the detection threshold for HOMER. To identify cluster-specific motif enrichment in our ATAC-seq clusters we ran HOMER for each cluster using the union set of dynamic peaks as our background (-bg) set with the options -size given –h. The resulting heat map was generated by combining the significant (*P* < 0.05) motifs for each cluster and plotting the associated *P* value. For motif distance measuring, we overlapped SOX9-bound opening peaks with known AP1 and SOX motifs curated by HOMER (mm10-191020) and measured the distance from SOX motifs to the closest AP1 motifs with Bedtools. For footprint analysis, we used HINT-ATAC^57^ with our 79 motif clusters as the input as well. For transcription factor motif variability score analysis, we ran ChromVAR^31^ (1.18.0) on the dynamic peaks for differential chromatin accessibility across our 79 motif clusters to find the top variable motifs in dynamic peaks. We further used ChromVAR to calculate the motif deviation scores over time at the top variable motifs.

#### Illustrations

Schematics were prepared using BioRender and Adobe Illustrator (v. 26.0.1).

#### Statistics and reproducibility

No statistical methods were used to pre-determine sample sizes, but our sample sizes are similar to those reported in previous publications^14,16,46^. No data points were excluded. Upon collection, mice with the same genetic background were randomly allocated to genomic or immunofluorescence experiments. Data collection and analysis were not performed blind to the conditions of the experiments as the mice appears phenotypical after SOX9 induction. All immunofluorescence experiments were repeated three times with samples collected from different mice. All co-immunoprecipitation and immunoblot experiments were repeated twice with samples collected on different days. The statistics in Fig. 5g and Extended Data Fig. 1d were analysed with two-tailed *t*-test on the GraphPad Prism (9.0). Data distribution was assumed to be normal, but this was not formally tested. All the error bars are mean ± s.d. *P < 0.05, **P < 0.01, ***P < 0.001 and ****P < 0.0001.

### Resource availability

#### Lead contact

Further information and requests for resources and reagents should be directed to and will be fulfilled by the lead contact, E.F. (fuchslb@rockefeller.edu).

#### Materials availability

Will be provided upon request and available upon publication.

### Reporting summary

Further information on research design is available in the Nature Portfolio Reporting Summary linked to this article.

## Online content

Any methods, additional references, Nature Portfolio reporting summaries, source data, extended data, supplementary information, acknowledgements, peer review information; details of author contributions and competing interests; and statements of data and code availability are available at 10.1038/s41556-023-01184-y.

## Supplementary information

Reporting Summary

Supplementary Table 1 Gene expression matrix across timepoints. Supplementary Table 3. SOX9-specific interactors from BioID. Supplementary Table 4. Primers for genotyping and quantitative PCR.

Supplementary Table 2 GO results of ATAC clusters (binomial is used to calculate the significance).

## Data Availability

All data that support the findings of this study are available within the paper and its supplementary files. Sequencing data that support the findings of this study have been deposited in the Gene Expression Omnibus under accession code GSE208072. Previously published RNA-seq data from BCC, SCC and normal EpdSCs that were re-analysed here are available under accession code GSE152487. Source data are provided with this paper. All other data supporting the findings of this study are available from the corresponding author on reasonable request.

## Source data

Source Data Table 1 Statistical source data.
Source Data Extended Data Fig./Table 1 Unprocessed western blots.

## Code availability

All bioinformatic analysis tools and pipelines used in this study are documented in the method section. Codes are available from the corresponding author upon reasonable request.
