# Supplementary material for: The pioneer factor SOX9 competes for epigenetic factors to switch stem cell fates
Source: Nat Cell Biol. 2023 Jul 24;25(8):1185–95. doi: 10.1038/s41556-023-01184-y (PMC10415178; doi:10.1038/s41556-023-01184-y)
Supplement: Supplementary file 1 — Reporting Summary [file 41556_2023_1184_MOESM1_ESM.pdf]

nature portfolio

Corresponding author(s): Elaine Fuchs, PhD

Last updated by author(s): May 8, 2023

Reporting Summary

Nature Portfolio wishes to improve the reproducibility of the work that we publish. This form provides structure for consistency and transparency in reporting. For further information on Nature Portfolio policies, see our Editorial Policies and the Editorial Policy Checklist.

Statistics

For all statistical analyses, confirm that the following items are present in the figure legend, table legend, main text, or Methods section.

n/a	Confirmed	
☐	☒	The exact sample size (*n*) for each experimental group/condition, given as a discrete number and unit of measurement
☐	☒	A statement on whether measurements were taken from distinct samples or whether the same sample was measured repeatedly
☐	☒	The statistical test(s) used AND whether they are one- or two-sided<br>*Only common tests should be described solely by name; describe more complex techniques in the Methods section.*
☐	☒	A description of all covariates tested
☐	☒	A description of any assumptions or corrections, such as tests of normality and adjustment for multiple comparisons
☐	☒	A full description of the statistical parameters including central tendency (e.g. means) or other basic estimates (e.g. regression coefficient) AND variation (e.g. standard deviation) or associated estimates of uncertainty (e.g. confidence intervals)
☐	☒	For null hypothesis testing, the test statistic (e.g. *F*, *t*, *r*) with confidence intervals, effect sizes, degrees of freedom and *P* value noted<br>*Give P values as exact values whenever suitable.*
☒	☐	For Bayesian analysis, information on the choice of priors and Markov chain Monte Carlo settings
☒	☐	For hierarchical and complex designs, identification of the appropriate level for tests and full reporting of outcomes
☒	☐	Estimates of effect sizes (e.g. Cohen's *d*, Pearson's *r*), indicating how they were calculated

Our web collection on statistics for biologists contains articles on many of the points above.

Software and code

Policy information about availability of computer code

Data collection	BD FACSDiva (v. 8.0) for FACS sorting; Illumina FASTQ Toolkit for sequencing.
Data analysis	Skewer (v0.2.2), Salmon (v1.4.0), Tximport (v1.12.3), R (v 3.6.1), DESeq2 (v 1.16.1), PEPATAC (v0.10.3), MACS2 (v2.2.7.1), Bowtie2 (v2.2.9), Picard (v2.3.0), Samtools (v1.3.1), Deeptools (v3.1.2), Integrative Genomics Viewer, SEACR, epic2 (v0.0.52) , GSEA (4.1.0), profileplyr(v1.4.3) HOMER (v4.10), JASPAR (2018), HINT-ATAC, ChromVar(v1.18.0), Adobe Illustrator (v26.0.1)

For manuscripts utilizing custom algorithms or software that are central to the research but not yet described in published literature, software must be made available to editors and reviewers. We strongly encourage code deposition in a community repository (e.g. GitHub). See the Nature Portfolio guidelines for submitting code & software for further information.

Data

Policy information about availability of data

All manuscripts must include a data availability statement. This statement should provide the following information, where applicable:

- Accession codes, unique identifiers, or web links for publicly available datasets
- A description of any restrictions on data availability
- For clinical datasets or third party data, please ensure that the statement adheres to our policy

The datasets generated during and/or analysed during the current study are available in the GEO repository, GSE208072, and the supplementary data.

Human research participants

Policy information about studies involving human research participants and Sex and Gender in Research.

Reporting on sex and gender	No human research participants were used.
Population characteristics	See above
Recruitment	See above
Ethics oversight	See above

Note that full information on the approval of the study protocol must also be provided in the manuscript.

Field-specific reporting

Please select the one below that is the best fit for your research. If you are not sure, read the appropriate sections before making your selection.

☒ Life sciences ☐ Behavioural & social sciences ☐ Ecological, evolutionary & environmental sciences

For a reference copy of the document with all sections, see nature.com/documents/nr-reporting-summary-flat.pdf

Life sciences study design

All studies must disclose on these points even when the disclosure is negative.

Sample size	Sample sizes are similar to those reported in previous publications using a similar approach (Naik and Larsen et al. Nature, 2017, Adam et al. Nature, 2015)
Data exclusions	No data was excluded for the analysis.
Replication	For our genomic experiments, each sample consisted of 3-4 mice per time point. Replicative analysis showed strong correlations for every one of our data types.
Randomization	K14rtTA+;TRE-SOX9+ mice were confirmed via early postnatal PCR genotyping. K14rtTA+;TRE-SOX9+ mice were housed with at least 2 K14rtTA + only males. Upon harvest, samples were separated into experimental and control samples and randomly allocated to genomic and immunfluoresence experiments.
Blinding	Blinding was not possible or relevant in our study. Our mice were phenotypic upon harvesting. Additionally, we conducted a time-course study with D0 as control.

Reporting for specific materials, systems and methods

We require information from authors about some types of materials, experimental systems and methods used in many studies. Here, indicate whether each material, system or method listed is relevant to your study. If you are not sure if a list item applies to your research, read the appropriate section before selecting a response.

Materials & experimental systems

n/a	Involved in the study	
☐	☒	Antibodies
☐	☒	Eukaryotic cell lines
☒	☐	Palaeontology and archaeology
☐	☒	Animals and other organisms
☒	☐	Clinical data
☒	☐	Dual use research of concern

Methods

n/a	Involved in the study	
☐	☒	ChIP-seq
☐	☒	Flow cytometry
☒	☐	MRI-based neuroimaging

Antibodies

Antibodies used	Ly6A/E-APCCy7,BioLegend,cat#108126<br>CD49f-PECy7,BioLegend,cat#313622<br>CD34-Alexa660,Invitrogen,cat#50-0341-82

CD45-biotin,BioLegend,cat#103104
CD31-biotin,BioLegend,cat#102404
CD140a-biotin,BioLegend,cat#135910
CD117-biotin,BioLegend,cat#105804
Streptavidin-FITC,BioLegend,cat#405202
TruStain FcX,BioLegend,cat#101320
SOX9,Millipore,cat#ab5535
MYC-tag,Cell Signaling,cat#71D10
MLL3/4,Wysocka Lab,cat#N/A
totalH3,Active Motif,cat#39763
H3K27ac,Active Motif,cat#39133
H3K4me1,Cell Signaling,cat#D1A9
SOX9,Abcam,cat#ab185966
ITGA6,BD,cat#555734
KRT14,BioLegend,cat#906004
KRT10,Fuchs Lab,cat#N/A
EpCAM,Abcam,cat#ab71916
KRT6,Fuchs Lab,cat#N/A
RUNX1,Abcam,cat#ab229482
GATA3,Invitrogen,cat#14-9966-82
HA-tag,Cell Signaling,cat#C29F4
GFP,Fuchs Lab,cat#N/A
RFP,ChromoTek,cat#5F8
MYC-tag,Cell Signaling,cat#71D10
MYC-tag,Cell Signaling,cat#9B11
beta-Actin,Cell Signaling,cat#8H10D10
MLL4,Santa Cruz Biotechnology,cat#sc-293217
MYC-tag,Cell Signaling,cat#2276S
IgG,Cell Signaling,cat#2729S
ARID1a,Cell Signaling,cat#12354S
ARID1a, Abcam,cat#ab182560
JUN,Cell Signaling,cat#3753S
BRG1,EpiCypher,cat#13-2002
Secondary (all with donkey as host):
AF488-Rabbit,Thermo Fisher,A-21206
AF488-Chicken,Thermo Fisher,A78948
AF546-Rabbit,Thermo Fisher,A10040
AF546-Rat,Thermo Fisher,A78947

Validation

The fluorophore conjugated antibodies from and Biolegend were validated for flow cytometry:
Ly6A/E-APCCy7,BioLegend,cat#108126
CD49f-PECy7,BioLegend,cat#313622
CD34-Alexa660,Invitrogen,cat#50-0341-82
CD45-biotin,BioLegend,cat#103104
CD31-biotin,BioLegend,cat#102404
CD140a-biotin,BioLegend,cat#135910
CD117-biotin,BioLegend,cat#105804
Streptavidin-FITC,BioLegend,cat#405202
TruStain FcX,BioLegend,cat#101320

The following antibodies are validated with western blot:
SOX9,Millipore,cat#ab5535
MYC-tag,Cell Signaling,cat#71D10
MYC-tag,Cell Signaling,cat#9B11
beta-Actin,Cell Signaling,cat#8H10D10
MLL4,Santa Cruz Biotechnology,cat#sc-293217 (also validated with KO in this study)

The following antibodies are validated with immunofluorescence:
SOX9,Abcam,cat#ab185966
ITGA6,BD,cat#555734
KRT14,BioLegend,cat#906004
KRT10,Fuchs Lab,cat#N/A
EpCAM,Abcam,cat#ab71916
KRT6,Fuchs Lab,cat#N/A
RUNX1,Abcam,cat#ab229482
GATA3,Invitrogen,cat#14-9966-82
HA-tag,Cell Signaling,cat#C29F4
GFP,Fuchs Lab,cat#N/A
RFP,ChromoTek,cat#5F8
all secondary antibodies

The following antibodies are validated for ChIP:
MLL3/4,Wysocka Lab,cat#N/A
totalH3,Active Motif,cat#39763
H3K27ac,Active Motif,cat#39133
H3K4me1,Cell Signaling,cat#D1A9

ARID1a,Cell Signaling,cat#12354S
ARID1a, Abcam,cat#ab182560
JUN,Cell Signaling,cat#3753S
BRG1,EpiCypher,cat#13-2002

The following antibodies are validated for coIP:
MYC-tag,Cell Signaling,cat#2276S
IgG,Cell Signaling,cat#2729S

Eukaryotic cell lines

Policy information about cell lines and Sex and Gender in Research

Cell line source(s)	Mouse keratinocyte cell lines from K14rtTA+ or K14rtTA+;TRE-SOX9+ mice. The sex is not available from the cell lines.
Authentication	The cell lines used for this study were generated within the Fuchs laboratory and confirmed by PCR genotyping.
Mycoplasma contamination	These specific Cell lines were not tested for mycoplasma but our lab routinely performs mycoplasma contamination checks of randomly selected lines throughout the year.
Commonly misidentified lines (See ICLAC register)	No cell lines in the ICLAC database were used.

Animals and other research organisms

Policy information about studies involving animals; ARRIVE guidelines recommended for reporting animal research, and Sex and Gender in Research

Laboratory animals	Mice {Mus musculus} from 2 transgenic mouse lines: TRE-MYC-SOX9;K14rtTA and K14rtTA only. 3-4 male mice at age 3-9 weeks were pooled per time point analyzed.<br>For skin grafts, 6-8 week old female Nude mice were used as recipients for P0 male mouse skin engraftment.
Wild animals	The study did not involve wild animals.
Reporting on sex	In order to maximize cell numbers and minimize variation due to sex, we used male mice for all experiments. Male mice were generally larger enabling more surface area of the skin to harvest EpdSCs.
Field-collected samples	The study did not involve samples collected from the field.
Ethics oversight	All experimental procedures were conducted with accordance and approval of the Institutional Animal Care and Use Committee (IACUC) – approved protocols at the Rockefeller University (20012-H and 20066-H).

Note that full information on the approval of the study protocol must also be provided in the manuscript.

ChIP-seq

Data deposition

☒ Confirm that both raw and final processed data have been deposited in a public database such as GEO.

☒ Confirm that you have deposited or provided access to graph files (e.g. BED files) for the called peaks.

Data access links<br>*May remain private before publication.*	https://www.ncbi.nlm.nih.gov/geo/query/acc.cgi?acc=GSE208072
Files in database submission	ATAC:<br>Sample 1-D0_ATAC_rep1<br>Sample 2-D0_ATAC_rep2<br>Sample 3-W1_ATAC_rep1<br>Sample 4-W1_ATAC_rep2<br>Sample 5-W2_ATAC_rep1<br>Sample 6-W2_ATAC_rep2<br>Sample 7-W6_ATAC_rep1<br>Sample 8-W6_ATAC_rep2<br>Sample 9-W12_ATAC_rep1<br>Sample 10-W12_ATAC_rep2<br>Sample 11-cultured_WT_dox_rep1<br>Sample 12-cultured_WT_nodox_rep1<br>Sample 13-cultured_noHMG_dox_rep1<br>Sample 14-cultured_noTA_dox_rep1<br>Sample 15-cultured_WT_dox_rep2<br>Sample 16-cultured_WT_nodox_rep2

Sample 17-cultured_noHMG_dox_rep2
Sample 18-cultured_noTA_dox_rep2
Sample 19-cultured_K14AFOS_dox_rep1
Sample 20-cultured_K14AFOS_dox_rep2
Sample 21-cultured_K14SOX9AFOS_dox_rep1
Sample 22-cultured_K14SOX9AFOS_dox_rep2
Sample 23-cultured_K14SOX9ARID1a_dox_rep1
Sample 24-cultured_K14SOX9ARID1a_dox_rep2
MINT-ChIP:
Sample 1-D0_H3K4me1_rep1_rep2
Sample 2-D0_H3K27ac_rep1_rep2
Sample 3-D0_totalH3_rep1_rep2
Sample 4-W1_H3K4me1_rep1_rep2
Sample 5-W1_H3K27ac_rep1_rep2
Sample 6-W1_totalH3_rep1_rep2
Sample 7-W2_H3K4me1_rep1_W12_H3K4me1_rep1_rep2
Sample 8-W2_H3K4me1_rep2
Sample 9-W2_H3K27ac_rep1_W12_H3K27ac_rep1_rep2
Sample 10-W2_H3K27ac_rep2
Sample 11-W2_totalH3_rep1_W12_totalH3_rep1_rep2
Sample 12-W2_totalH3_rep2
Sample 13-W6_H3K4me1_rep1
Sample 14-W6_H3K4me1_rep2
Sample 15-W6_H3K27ac_rep1
Sample 16-W6_H3K27ac_rep2
Sample 17-W6_totalH3_rep1
Sample 18-W6_totalH3_rep2
Bulk RNA-Seq:
Sample 1-RNA_D0_rep1
Sample 2-RNA_D0_rep2
Sample 3-RNA_W1_rep1
Sample 4-RNA_W1_rep2
Sample 5-RNA_W2_rep1
Sample 6-RNA_W2_rep2
Sample 7-RNA_W6_rep1
Sample 8-RNA_W6_rep2
Sample 9-RNA_W12_rep1
Sample 10-RNA_W12_rep2
Sample 11-RNA_SOX9neg_rep1
Sample 12-RNA_SOX9neg_rep2
Sample 13-RNA_SOX9pos_rep1
Sample 14-RNA_SOX9pos_rep2
Cut-and-Run:
Sample 1-SOX9CNR_D0_rep1
Sample 2-SOX9CNR_D0_rep2
Sample 3-SOX9CNR_W1_rep1
Sample 4-SOX9CNR_W1_rep2
Sample 5-SOX9CNR_W2_rep1
Sample 6-SOX9CNR_W2_rep2
Sample 7-SOX9CNR_W6_rep1
Sample 8-SOX9CNR_W6_rep2
Sample 9-SOX9CNR_W12_rep1
Sample 10-SOX9CNR_W12_rep2
Sample 11-mllCNR_D0_rep1
Sample 12-mllCNR_D0_rep2
Sample 13-mllCNR_W1_rep1
Sample 14-mllCNR_W1_rep2
Sample 15-mllCNR_W2_rep1
Sample 16-mllCNR_W2_rep2
Sample 17-mllCNR_WTSOX9_nodox_rep1
Sample 18-mllCNR_noHMG_dox_rep1
Sample 19-mllCNR_noTA_dox_rep1
Sample 20-mycCNR_WTSOX9_dox_rep1
Sample 21-mycCNR_WTSOX9_nodox_rep1
Sample 22-mycCNR_noHMG_dox_rep1
Sample 23-mycCNR_noTA_dox_rep1
Sample 24-mllCNR_WTSOX9_nodox_rep2
Sample 25-mllCNR_noHMG_dox_rep2
Sample 26-mllCNR_noTA_dox_rep2
Sample 27-mycCNR_WTSOX9_dox_rep2
Sample 28-mycCNR_WTSOX9_nodox_rep2
Sample 29-mycCNR_noHMG_dox_rep2
Sample 30-mycCNR_noTA_dox_rep2
Sample 31-mllCNR_WTSOX9_dox_rep1
Sample 32-mllCNR_WTSOX9_dox_rep2
Sample 33-JunCNR_WTSOX9_nodox_rep1

Sample 34-JunCNR_WTSOX9_dox_rep1
Sample 35-JunCNR_noHMG_dox_rep1
Sample 36-Arid1aCNR_WTSOX9_nodox_rep1
Sample 37-Arid1aCNR_WTSOX9_dox_rep1
Sample 38-Arid1aCNR_noHMG_dox_rep1
Sample 39-BRG1CNR_WTSOX9_nodox_rep1
Sample 40-BRG1CNR_WTSOX9_dox_rep1
Sample 41-BRG1CNR_noHMG_dox_rep1

Genome browser session (e.g. UCSC)

Available upon reasonable request. All processed bigwig files are provided in GEO for visualization.

Methodology

Replicates

Each data set was replicated twice at independent times with different cohorts of mice. Experimental replicates were highly concordant and displayed within the extended data.

Sequencing depth

Sample,Aligned Reads,Paired/Single End reads
D0_Rep1_ATAC,43858222,50bp Paired-end
D0_Rep2_ATAC,39846438,50bp Paired-end
W1_Rep1_ATAC,34846318,50bp Paired-end
W1_Rep2_ATAC,37261602,50bp Paired-end
W2_Rep1_ATAC,18838504,50bp Paired-end
W2_Rep2_ATAC,13723684,50bp Paired-end
W6_Rep1_ATAC,30156910,50bp Paired-end
W6_Rep2_ATAC,19672580,50bp Paired-end
W12_Rep1_ATAC,72898350,50bp Paired-end
W12_Rep2_ATAC,19433948,50bp Paired-end
D0_Rep1_H3K27ac,3841033,50bp Paired-end
D0_Rep2_H3K27ac,3121333,50bp Paired-end
W1_Rep1_H3K27ac,711249,50bp Paired-end
W1_Rep2_H3K27ac,3074037,50bp Paired-end
W2_Rep1_H3K27ac,1030516,50bp Paired-end
W2_Rep2_H3K27ac,3928882,50bp Paired-end
W6_Rep1_H3K27ac,7956088,50bp Paired-end
W6_Rep2_H3K27ac,1455927,50bp Paired-end
W12_Rep1_H3K27ac,1258550,50bp Paired-end
W12_Rep2_H3K27ac,1403434,50bp Paired-end
D0_Rep1_H3K4me1,24592016,50bp Paired-end
D0_Rep2_H3K4me1,22063883,50bp Paired-end
W1_Rep1_H3K4me1,6587117,50bp Paired-end
W1_Rep2_H3K4me1,9550526,50bp Paired-end
W2_Rep1_H3K4me1,6041330,50bp Paired-end
W2_Rep2_H3K4me1,10653668,50bp Paired-end
W6_Rep1_H3K4me1,27215545,50bp Paired-end
W6_Rep2_H3K4me1,8697635,50bp Paired-end
W12_Rep1_H3K4me1,6916330,50bp Paired-end
W12_Rep2_H3K4me1,8388474,50bp Paired-end
D0_Rep1_Total H3,37680307,50bp Paired-end
D0_Rep2_Total H3,32670127,50bp Paired-end
W1_Rep1_Total H3,12964069,50bp Paired-end
W1_Rep2_Total H3,13337757,50bp Paired-end
W2_Rep1_Total H3,9340777,50bp Paired-end
W2_Rep2_Total H3,58641446,50bp Paired-end
W6_Rep1_Total H3,40744095,50bp Paired-end
W6_Rep2_Total H3,26969269,50bp Paired-end
W12_Rep1_Total H3,8736431,50bp Paired-end
W12_Rep2_Total H3,11271301,50bp Paired-end
D0_Rep1_SOX9_CNR,20174088,50bp Paired-end
D0_Rep2_SOX9_CNR,19645106,50bp Paired-end
W1_Rep1_SOX9_CNR,23997258,50bp Paired-end
W1_Rep2_SOX9_CNR,370168,50bp Paired-end
W2_Rep1_SOX9_CNR,9768786,50bp Paired-end
W2_Rep2_SOX9_CNR,7253702,50bp Paired-end
W6_Rep1_SOX9_CNR,30653330,50bp Paired-end
W6_Rep2_SOX9_CNR,2028772,50bp Paired-end
W12_Rep1_SOX9_CNR,5090868,50bp Paired-end
W12_Rep2_SOX9_CNR,20178366,50bp Paired-end
D0_Rep1_Mll_CNR,7024229,50bp Paired-end
D0_Rep2_Mll_CNR,3703596,50bp Paired-end
W1_Rep1_Mll_CNR,10820527,50bp Paired-end
W1_Rep2_Mll_CNR,6310082,50bp Paired-end
W2_Rep1_Mll_CNR,5048765,50bp Paired-end
W2_Rep2_Mll_CNR,5542309,50bp Paired-end

	WTSOX9_Nodox_Rep1_Mll_CNR,10946976,50bp Paired-end<br>noHMG_Dox_Rep1_Mll_CNR,6801070,50bp Paired-end<br>noTA_Dox_Rep1_Mll_CNR,7902664,50bp Paired-end<br>WTSOX9_Nodox_Rep1_Myc_CNR,3199032,50bp Paired-end<br>noHMG_Dox_Rep1_Myc_CNR,2733154,50bp Paired-end<br>WTSOX9_Dox_Rep1_Myc_CNR,3983006,50bp Paired-end<br>noTA_Dox_Rep1_Myc_CNR,3532914,50bp Paired-end
Antibodies	SOX9,Millipore,cat#ab5535<br>MLL3/4,Wysocka Lab,cat#N/A<br>totalH3,Active Motif,cat#39763<br>H3K27ac,Active Motif,cat#39133<br>H3K4me1,Cell Signaling,cat#D1A9<br>MYC-tag,Cell Signaling,cat#2276S<br>IgG,Cell Signaling,cat#2729S<br>ARID1a,Cell Signaling,cat#12354S<br>JUN,Cell Signaling,cat#3753S<br>BRG1,EpiCypher,cat#13-2002
Peak calling parameters	ATAC-Seq: Replicate BAM files were merged, and peak calling was performed using Model-based Analysis of ChIP-Seq 2 (MACS2) with the option of "--keep-dup all" to keep duplicates generated during the combining of experimental replicates.<br><br>H3K27ac: Replicate BAM files were merged with keep-duplicate option and MACS2 called with standard parameters and total H3 as input.<br><br>H3K4me1: Replicate BAM files were merged and coverted to BEDPE. --treatment was sample and -c was Total H3.<br><br>Cut-and-Run: D0 to W12 SOX9 peaks were called using SEACR37 from bedGraph files generated from RPKM normalized Bigwig files (bigWigToBedGraph, UCSC Tools) using stringent setting and a numeric threshold of 0.01. Peaks were further filtered to have peaks scores > 1800 for a set of high confident peaks.
Data quality	Data was checked for high correlations between replicate samples and enrichment for known regulatory regions such as TSS for ATAC, and H3K27ac. SOX9 CNR was assayed by unbiased motif enrichment at called peaks. MLL3/4 CNR was assayed by enrichment at enhancer elements relative to promoter regions.
Software	Skewer (v0.2.2), R (v 3.6.1), MACS2 (v2.2.7.1), Bowtie2 (v2.2.9), Picard (v2.3.0), Samtools (v1.3.1), Deeptools (v3.1.2), Integrative Genomics Viewer, SEACR, epic2 (v0.0.52) , GSEA, profileplyr(v1.4.3) HOMER (v4.10), JASPAR (2018), HINT-ATAC, ChromVar(v1.18.0)

Flow Cytometry

Plots

Confirm that:

☒ The axis labels state the marker and fluorochrome used (e.g. CD4-FITC).

☒ The axis scales are clearly visible. Include numbers along axes only for bottom left plot of group (a 'group' is an analysis of identical markers).

☒ All plots are contour plots with outliers or pseudocolor plots.

☒ A numerical value for number of cells or percentage (with statistics) is provided.

Methodology

Sample preparation	Backskin of K14rtTA+;TRE-SOX9; and K14rtTA+ only mice were harvested and subjected to 0.25% trypsin/EDTA dissociation for 1 hour at 37C. After neutralization with FACS buffer cell suspension was strained, centrifuged and resuspended prior to antibody staining. A cocktail of antibodies for surface markers was prepared at predetermined concentrations in FACS buffer followed by washing and resuspension in secondary antibody. The cells were washed and resuspended again in FACS buffer containing 100 ng/mL of DAPI and filtered again through 70 uM filter caps before FACS.
Instrument	BD Biosciences FACSAria equipped with FACSDiva software for sorting, BD Biosciences FACS Fortessa with FACSDiva software for analysis.
Software	FACSDiva 8.0 for operating the sorter or analyzer.
Cell population abundance	Post sorting of the samples was routinely performed and consistently showed greater than 90% purity of the isolated populations.
Gating strategy	Single cell suspensions of harvested skin cells were first gated on ITGA6+ and Lineage negative (CD140a-,CD45-,CD117-,CD31-) followed by enrichment for EpdSC by gating on cells which were Ly6a+, CD34-, while HFSC were Ly6a-,CD34+. Please see extended data figure 2.

☒ Tick this box to confirm that a figure exemplifying the gating strategy is provided in the Supplementary Information.
