# Supplementary material for: The pioneer factor SOX9 competes for epigenetic factors to switch stem cell fates
Source: Nat Cell Biol. 2023 Jul 24;25(8):1185–95. doi: 10.1038/s41556-023-01184-y (PMC10415178; doi:10.1038/s41556-023-01184-y)
Supplement: Source Data Extended Data Fig./Table 1 — Unprocessed western blots. [file 41556_2023_1184_MOESM5_ESM.pdf]

Extended Data Fig.5g Full Blot

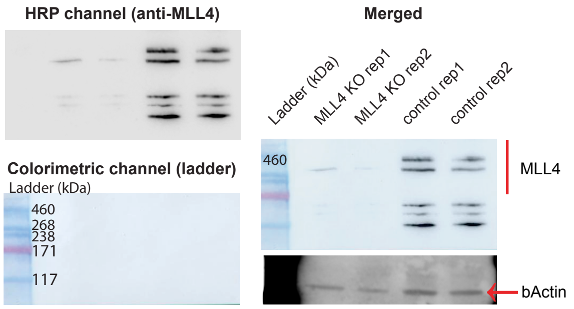

Extended Data Fig.5h Full Blot

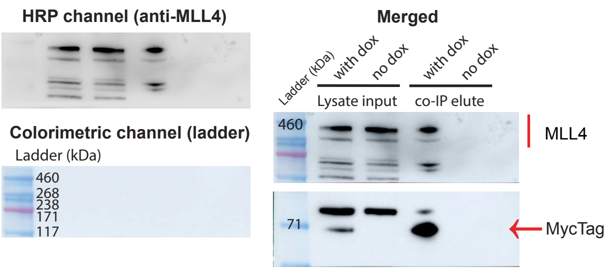

Extended Data Fig.7d Full Blot

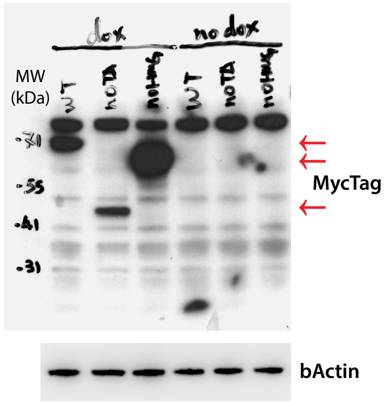

Extended Data Fig.7e Full Blot

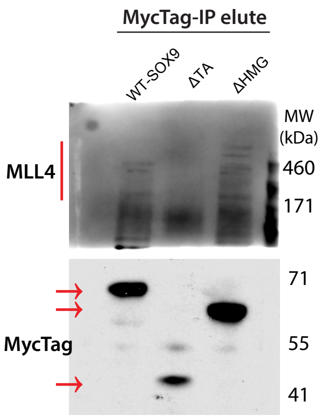

Extended Data Fig.7i Full Blot

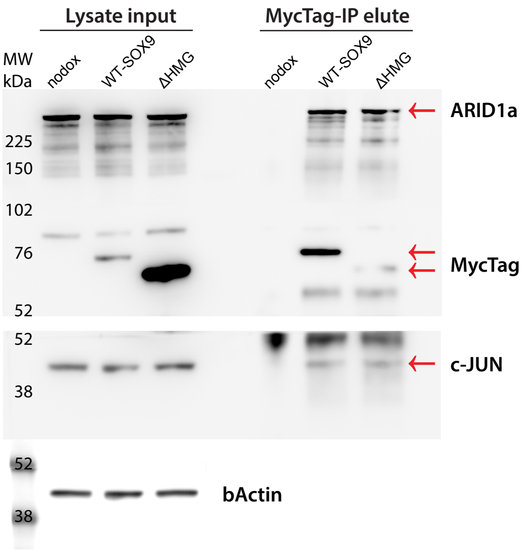

Extended Data Fig.7j Full Blot

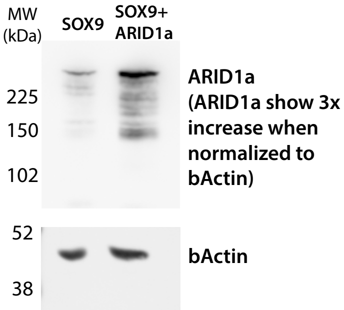

SOX9: K14rtTA;TRE-myc-SOX9
SOX9+ARID1a: K14rtTA; TRE-myc-SOX9; TRE-ARID1a
